# Characterization of multiple binding sites on microtubule associated protein 2c recognized by dimeric and monomeric 14‐3‐3ζ

**DOI:** 10.1111/febs.17405

**Published:** 2025-01-29

**Authors:** Séverine Jansen, Subhash Narasimhan, Paula Cabre Fernandez, Lucia Iľkovičová, Aneta Kozeleková, Kateřina Králová, Jozef Hritz, Lukáš Žídek

**Affiliations:** ^1^ Central European Institute of Technology Masaryk University Brno Czech Republic; ^2^ National Centre for Biomolecular Research, Faculty of Science Masaryk University Brno Czech Republic; ^3^ Research Institute Sant Pau Barcelona Spain; ^4^ Department of Chemistry, Faculty of Science Masaryk University Brno Czech Republic

**Keywords:** 14‐3‐3 proteins, extracellular signal‐regulated kinase 2, microtubule‐associated protein, nuclear magnetic resonance, protein kinase A

## Abstract

Microtubule associated protein 2 (MAP2) interacts with the regulatory protein 14‐3‐3ζ in a cAMP‐dependent protein kinase (PKA) phosphorylation dependent manner. Using selective phosphorylation, calorimetry, nuclear magnetic resonance, chemical crosslinking, and X‐ray crystallography, we characterized interactions of 14‐3‐3ζ with various binding regions of MAP2c. Although PKA phosphorylation increases the affinity of MAP2c for 14‐3‐3ζ in the proline rich region and C‐terminal domain, unphosphorylated MAP2c also binds the dimeric 14‐3‐3ζ via its microtubule binding domain and variable central domain. Monomerization of 14‐3‐3ζ leads to the loss of affinity for the unphosphorylated residues. In neuroblastoma cell extract, MAP2c is heavily phosphorylated by PKA and the proline kinase ERK2. Although 14‐3‐3ζ dimer or monomer do not interact with the residues phosphorylated by ERK2, ERK2 phosphorylation of MAP2c in the C‐terminal domain reduces the binding of MAP2c to both oligomeric variants of 14‐3‐3ζ.

## Graphical abstract

Microtubule associated protein 2c (MAP2c) interacts with the regulatory protein 14‐3‐3ζ in a phosphorylation‐dependent manner. In its unphosphorylated state, MAP2c binds 14‐3‐3ζ via its microtubule binding domain and its central region. However, upon phosphorylation by cAMP‐dependent protein kinase (PKA), 14‐3‐3ζ preferentially binds to the phosphorylated residues in the C‐terminal domain and central region. Notably, monomeric 14‐3‐3ζ only binds phosphorylated residues. Additionally, ERK2 phosphorylation reduces the affinity of 14‐3‐3ζ for the phosphorylated residue in the C‐terminal domain.

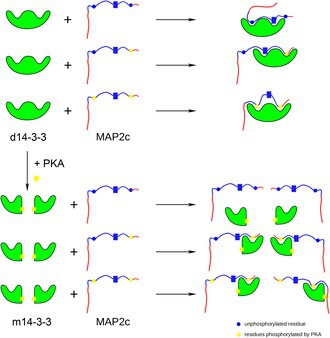

**Abbreviations**
CTD: C‐terminal domain
d14‐3‐3ζ: dimeric 14‐3‐3ζ
DSBU: disuccinimidyl dibutyric urea
ERK2: extracellular signal‐regulated kinase 2
ITC: isothermal titration calorimetry
m14‐3‐3ζ: monomeric 14‐3‐3ζ
MAP2: microtubule‐associated protein 2
MP‐MAP2c: MAP2c fully phosphorylated by PKA
MTBD: microtubule‐binding domain
NMR: nuclear magnetic resonance
NTD: N‐terminal domain
PAGE: polyacrylamide gel electrophoresis
PKA: cAMP‐dependent protein kinase A
PRR: proline‐rich region
SDS/PAGE: sodium dodecyl sulfate polyacrylamide gel electrophoresis
SP‐MAP2c: MAP2c phosphorylated selectively by PKA at Ser435
UP‐MAP2c: unphosphorylated MAP2c
VCR: variable central region

## Introduction

Cytoskeletal microtubule associated proteins (MAPs) regulate the stability and the dynamics of microtubules [1]. MAP2 and Tau are the main representants of MAPs in the brain; MAP2 proteins are mainly expressed in the dendrites, while Tau is mainly present in axons [2]. Both MAP2 and Tau belong to the class of intrinsically disordered proteins (IDPs), which lack a defined tertiary structure and exist in multiple quickly interconverting conformations [3, 4, 5, 6, 7]. MAP2 and Tau differ mostly in the N‐terminal region, containing the N‐terminal domain (NTD), the variable central region (VCR), and the proline‐rich region (PRR), while their C‐terminal region, containing the microtubule‐binding domain (MTBD) and the C‐terminal domain (CTD), is homologous [8, 9]. The MAP2 family is composed of two high molecular weight proteins, MAP2a and MAP2b, consisting of 1942 and 1830 amino acids in rat, respectively, and two low molecular weight variants, MAP2c and MAP2d, consisting of 467 and 498 amino acids, respectively, resulting from alternative splicing [8]. They differ mainly in the N‐terminal domain, where the high molecular weights MAP2s contain additional ~ 1300 amino acids. MAP2c is expressed mainly perinatally. Postnatally, its expression is restricted to the zones showing postnatal plasticity, such as the olfactory bulb, suggesting a role in neuronal development [2, 10].

MAP2c is highly phosphorylated *in vivo*, and its phosphorylation is regulated during neuronal development. The phosphorylation state is controlled by a precise balance of kinase and phosphatase activities [11, 12]. In neuroblastoma cell extracts, MAP2c is phosphorylated by cAMP‐dependent protein kinase A (PKA) and by proline‐directed kinases, such as extracellular signal‐regulated kinase 2 (ERK2), among other kinases [13]. The phosphorylation state of MAP2 controls its interaction with microtubules, regulating microtubule dynamics [14, 15, 16, 17, 18, 19], and with a variety of cytoplasmic proteins, such as plectin, SH3 domains of Fyn and Abl, and the regulatory 14‐3‐3 proteins [13, 20, 21, 22].

14‐3‐3 is a family of highly conserved ubiquitous proteins, expressed abundantly in the brain, and having important roles in neuronal development [23, 24]. The 14‐3‐3 family contains seven isoforms in mammals, β, γ, ɛ, ζ, θ, τ, and η, the 14‐3‐3ζ being one of the isoforms associated with microtubules in brain [25]. 14‐3‐3 interacts predominantly with phosphorylated residues located in the consensus sequences RSXpS/pTXP (mode I), RXXXpS/pTXP (mode II), and pS/pT(X1‐2)‐COOH (mode III) [26, 27, 28, 29]. In addition to the bound structures, binding pathways of individual phosphopeptides and the corresponding potential of mean force profiles toward 14‐3‐3ζ were determined by computational approaches [30]. However, binding between 14‐3‐3 and unphosphorylated proteins has also been shown [31].

14‐3‐3 proteins are mostly dimeric in the cells. Each monomer of 14‐3‐3 consists of nine α‐helices, forming an amphipathic groove binding the phosphorylated targets, while the dimer interface is formed by four N‐terminal α‐helices stabilized by multiple salt bridges and conserved hydrophobic interactions [26, 32, 33, 34, 35, 36].

Function of 14‐3‐3 proteins can be regulated by disruption of their dimeric structure. Phosphorylation of Ser58 located at the 14‐3‐3 dimer interface by several kinases such as PKA, PKB/Akt, PKC‐δ, MAPK/AKP2 [37, 38, 39, 40] leads to significant shift in dimer‐monomer equilibrium toward monomers (from dimerization *K*_D_ of 5 nm for the 14‐3‐3ζ wild‐type to 4 mm for 14‐3‐3ζ phosphorylated at Ser58) [41]. Monomerization of 14‐3‐3 directly affects cellular processes such as apoptosis/cell survival [42, 43, 44], axon outgrowth [45], and neurodegeneration [46, 47]. Therefore, presence of monomeric 14‐3‐3 proteins in the cell cannot be overlooked. Noteworthy, phosphomimicking mutations such as S58E/D proved to be unreliable approximation with regard to phosphorylation [42]. On the other hand, monomerization of 14‐3‐3 proteins can be also induced by point mutations of residues at the dimeric interface, as in the case of mutant 14‐3‐3ζ L12E/M78K (m14‐3‐3ζ) with properties resembling the phosphorylated 14‐3‐3ζ monomer [42, 48].

MAP2 interacts with 14‐3‐3 and participates in the formation of dendrites *in vivo* [49]. Dimeric 14‐3‐3ζ (d14‐3‐3ζ) binds to MAP2c and Tau at residues phosphorylated by PKA, as well as at unphosphorylated residues, located in the MTBD [21, 50, 51, 52] (note that sequences of all binding sites are completely identical in rat and human MAP2c). In contrast, m14‐3‐3ζ binding requires phosphorylation by PKA in the case of Tau [53, 54]. Furthermore, the affinity of Tau phosphorylated by PKA is significantly lower for m14‐3‐3ζ than for d14‐3‐3ζ [55].

As we previously determined important differences between Tau and MAP2c in binding to d14‐3‐3ζ, we decided to characterize the recognition of different MAP2c binding sites by monomeric and dimeric 14‐3‐3ζ. We analyzed the binding of MAP2c to the stable L12E/M78K monomeric mutant of 14‐3‐3ζ [41, 48] and to d14‐3‐3ζ. We showed that binding of MAP2c to d14‐3‐3ζ involves competition between phosphorylated and unphosphorylated residues, while m14‐3‐3ζ binds exclusively to the PKA phosphorylated residues of MAP2c. We also showed that, although 14‐3‐3ζ does not interact with ERK2 phosphorylated residues, ERK2 phosphorylation influences the binding of MAP2c to both d14‐3‐3ζ and m14‐3‐3ζ.

## Results

### Phosphorylation of MAP2c by PKA increases its affinity for 14‐3‐3ζ

The apparent dissociation constants (*K*_D_) and stoichiometry of the complexes of unphosphorylated and phosphorylated MAP2c with d14‐3‐3ζ and m14‐3‐3ζ were determined using isothermal titration calorimetry (ITC). Three samples of MAP2c were used: unphosphorylated MAP2c (UP‐MAP2c), MAP2c phosphorylated selectively at Ser435 (SP‐MAP2c) prepared by a short incubation with PKA, and MAP2c phosphorylated by PKA for a long time (MP‐MAP2c), resulting in almost complete phosphorylation at Ser184, Thr220, and Ser435 [21]. Multi‐site fitting models failed to distinguish multiple dissociation constants. Therefore, a simple model assuming presence of *n* binding sites with the same affinity was used to quantify the ITC data. The results are shown in Fig. S1 and summarized in Table 1.

**Table 1:** Results of ITC of UP‐MAP2c, SP‐MAP2c, and MP‐MAP2c titrated by d14‐3‐3ζ and m14‐3‐3ζ.

Protein in cell	*K*_D_ (μm)	*n*	∆*G* (kJ·mol^−1^)	∆*H* (kJ·mol^−1^)	−*T*∆*S* (kJ·mol^−1^)
Titrated by d14‐3‐3ζ					
UP‐MAP2c	58 ± 6	1.93 ± 0.03	−24.4	11.4 ± 0.5	−35.8
SP‐MAP2c	74 ± 21	2.68 ± 0.12	−23.8	8.7 ± 1.0	−32.5
MP‐MAP2c	21 ± 3	1.99 ± 0.04	−26.9	5.6 ± 0.2	−32.5
Titrated by m14‐3‐3ζ					
UP‐MAP2c	226 ± 65	n.d.^a^	−20.9	n.d.^a^	n.d.^a^
SP‐MAP2c	102 ± 46	1.51 ± 0.1	−22.9	10.4 ± 2.8	−33.3
MP‐MAP2c	17 ± 5	1.31 ± 0.06	−27.3	3.2 ± 0.3	−30.5

^a^ Cannot be determined because *K*_D_ exceeds the MAP2c concentration in the cell.

We observed a high heat of dissociation for free m14‐3‐3ζ titrated in ITC buffer, suggesting that m14‐3‐3ζ is partially present as dimers at the high concentration necessary for ITC. The estimated dissociation constant of m14‐3‐3ζ was ~ 600 μm (data shown in Fig. S2). The heat of dissociation has been subtracted before evaluating the binding constant, but the value of *K*_D_ of the complex of UP‐MAP2c with m14‐3‐3ζ might be underestimated due to the presence of the m14‐3‐3ζ dimers.

The obtained dissociation constants show that phosphorylation of MAP2c at several residues increases affinity for both m14‐3‐3ζ and d14‐3‐3ζ. The binding was always driven by a large entropy gain, with a positive enthalpy change. However, phosphorylation reduces the unfavorable enthalpy change (by ~ 3 kJ·mol^−1^) for d14‐3‐3ζ. The *K*_D_ values of the MP‐MAP2c complexes with m14‐3‐3ζ and d14‐3‐3ζ were comparable.

The same applies also for the complexes of UP‐MAP2c and SP‐MAP2c. The overall stoichiometry (*n*) was 2 monomeric units of d14‐3‐3ζ per one molecule of UP‐MAP2c and MP‐MAP2c and ~ 2.7 for SP‐MAP2c. Values of *n* of 1.5 and 1.3, respectively, were obtained for SP‐MAP2c and MP‐MAP2c titrated by m14‐3‐3ζ. However, the proportion of dimers of m14‐3‐3ζ is close to 20% at 100 μm concentration, suggesting that the *K*_D_ and *n* values may be influenced by m14‐3‐3ζ dimerization at higher concentrations. The observed overall stoichiometries are compared with theoretical binding modes of multivalent ligands in Discussion.

In order to probe the effect of MAP2c phosphorylation on the 14‐3‐3ζ binding affinity more directly, we performed ITC with a MAP2c fragment containing only one site phosphorylated efficiently by PKA. The fragment consisted of residues 300–467, covering MTBD and CTD. The unphosphorylated fragment, referred to as UP‐(300–467), and the fragment phosphorylated by PKA on Ser435, referred to as SP‐(300–467), were titrated by d14‐3‐3ζ. The phosphorylation decreased apparent *K*_D_ from (56 ± 8) μm to (24 ± 6) μm (Fig. S3).

### Identification of 14‐3‐3ζ binding sites in MAP2c

Residue‐specific identification of 14‐3‐3ζ binding sites on MAP2c was based on the fact that peaks in NMR spectra of free intrinsically disordered MAP2c are sharp, whereas peaks of residues directly interacting with well‐ordered regions of 14‐3‐3ζ are substantially broadened in the MAP2c:14‐3‐3ζ complexes. Such an approach allowed us to resolve interactions of individual MAP2c residues and qualitatively compare their relative strengths. Examples of peak broadening in ^1^H,^15^N‐HSQC spectra are presented in Figs S4–S8. However, ^1^H,^15^N‐HSQC spectra of MAP2c do not provide sufficient resolution due to the peak overlap. Therefore, we monitored peak broadening in 3D HNCO spectra, which allowed us to distinguish most of the individual residues. Quantitative values of stoichiometry and *K*_D_ for the individual residues were not calculated because the peak heights depend not only on the amounts of the free and bound forms but also on the chemical shift changes and kinetics of the binding.

**Fig. 1:**
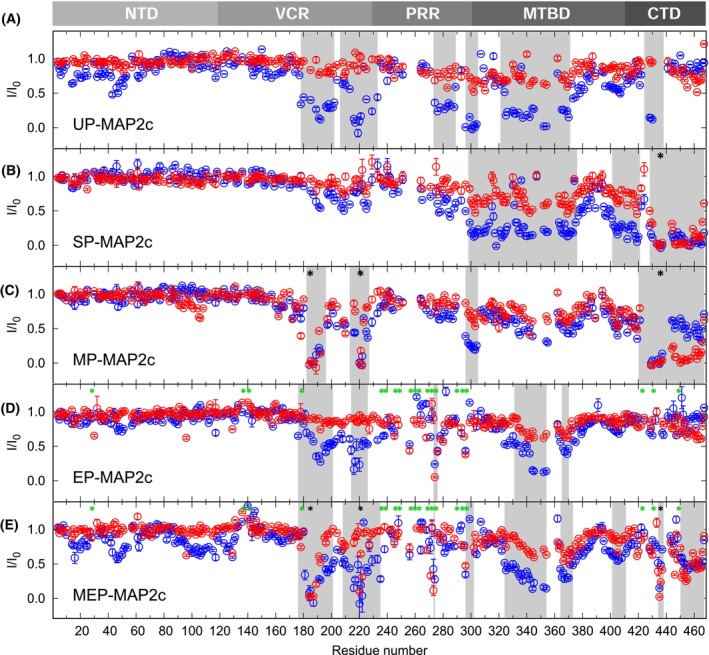
Identification of 14‐3‐3ζ binding sites in MAP2c followed by NMR peak broadening. Relative heights of the peaks in HNCO spectra upon addition of dimeric 14‐3‐3ζ (d14‐3‐3ζ, blue) or monomeric 4–3–3ζ (m14‐3‐3ζ, red) to unphosphorylated MAP2c (UP‐MAP2c, A), to MAP2c selectively phosphorylated by protein kinase (PKA) at Ser435 (SP‐MAP2c, B), to MAP2c fully phosphorylated by PKA (MP‐MAP2c, C), to MAP2c phosphorylated by extracellular signal‐regulated kinase 2 (ERK2, EP‐MAP2c, D), and to MAP2c phosphorylated by PKA and ERK2 (MEP‐MAP2c, E). The plotted values *I*/*I*_0_ are the ratios of peak heights in the presence and absence of 14‐3‐3ζ. Phosphorylation sites are indicated by black (PKA) and green (ERK2) asterisks. The regions interacting with 14‐3‐3ζ are underlined by gray rectangles. The error bars corresponding to the uncertainty of the measurement (calculated from the signal‐to‐noise ratio) document how representative the plotted data are. The reproducibility of the data showing significant effect on 14‐3‐3ζ binding was confirmed by independent experiments (14‐3‐3ζ concentration series, presented in Fig. 2, for UP‐MAP2c and MP‐MAP2c, ^1^H,^15^N‐HSQC for the binding fragment of SP‐MAP2c).

All HNCO spectra were recorded at 100 μm concentration of UP‐MAP2c, SP‐MAP2c, and MP‐MAP2c. Peak heights for the 200 μm concentration of d14‐3‐3ζ and m14–33ζ (expressed for the 14‐3‐3ζ monomer) are plotted in Fig. 1.

**Fig. 2:**
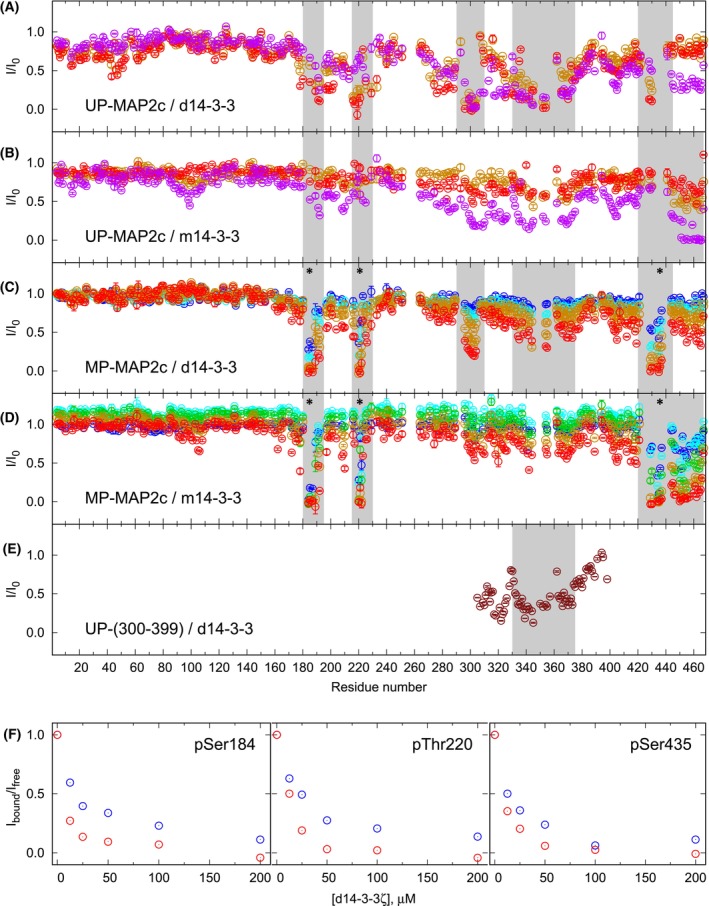
Titration of unphosphorylated MAP2c (UP‐MAP2c) and MAP2c fully phosphorylated by cAMP‐dependent Protein Kinase (MP‐MAP2c) with dimeric 14‐3‐3ζ (d14‐3‐3ζ) and monomeric 14‐3‐3ζ (m14‐3‐3ζ) followed by NMR. A and B, Reduction of peak heights in the HNCO spectra of 100 μm [^13^C,^15^N]‐UP‐MAP2c in the presence of 100 μm (orange), 200 μm (red, also presented in Fig. 1), and 800 μm (magenta) d14‐3‐3ζ (A, monomeric concentrations) and m14‐3‐3ζ (B). (C, D) Reduction of peak heights in the HNCO spectra of 100 μm [^13^C,^15^N]‐MP‐MAP2c in the presence of 12.5 μm (blue), 25 μm (cyan), 50 μm (green), 100 μm (orange), and 200 μm (red also presented in Fig. 1) d14‐3‐3ζ (C, monomeric concentrations) and m14‐3‐3ζ (D). PKA phosphorylation sites are indicated by black asterisks. (E) Reduction of peak heights in HNCO spectra of 350 μm [^13^C,^15^N]‐MAP2c Microtubule binding domain (UP‐(300–399), brown) upon addition of 350 μm d14‐3‐3ζ. The plotted *I*/*I*_0_ values are ratios of the peak heights of the UP‐MAP2c, MP‐MAP2c, or UP‐(300–399) complexes to the peak heights of free UP‐MAP2c, MP‐MAP2c, or UP‐(300–399). The error bars corresponding to the uncertainty of the measurement (calculated from the signal‐to‐noise ratio) document how representative the plotted data are. (F) Difference in the relative decrease of the peak heights of pSer184, pThr220, and pSer435 of 100 μm MP‐MAP2c in the presence of 0–200 μm d14‐3‐3ζ in ^1^H,^15^N‐HSQC (blue) and in HNCO (red) spectra measured at 950 MHz, indicating exchange contribution to the peak broadening. Decrease of peak heights was confirmed by a repeated measurement of ^1^H,^15^N‐HSQC at 750 MHz, showing similar results.

Only a marginal decrease in peak heights was observed for UP‐MAP2c in the presence of m14‐3‐3ζ, suggesting that UP‐MAP2c does not interact with m14‐3‐3ζ at 200 μm concentrations, and that the ITC data are biased by dimerization of m14‐3‐3ζ at high concentrations. At 800 μm, m14‐3‐3ζ shows substantial binding to UP‐MAP2c which confirms dimerization of m14‐3‐3ζ at this high concentration (Fig. 2B). In contrast, the peak heights decreased substantially (below 50%) in several regions in the presence of d14‐3‐3ζ, revealing the binding sites of UP‐MAP2c. The identified binding sites include residues 178–202 and 206–233 in VCR, 273–289, 296–305 and 321–371 in MTBD, and 424–438 in CTD. A small decrease in peak height (~ 40%) is also seen in NTD (residues 40–60), suggesting an interaction between the NTD and CTD of UP‐MAP2c. Using a MAP2c fragment containing the MTBD (residues 300–399; Fig. 2E), we observed a decrease of peak heights for residues 332–373, showing that the MTBD of UP‐MAP2c is able to bind even as an individual fragment to d14‐3‐3ζ. The selective phosphorylation of MAP2c at Ser435 induces binding of m14‐3‐3ζ to the phosphorylated serine, but also to the C‐terminal region (residues 428–467). In the case of d14‐3‐3ζ binding, the phosphorylation at Ser435 enhances binding to the same region as observed for m14‐3‐3ζ. It preserves the interaction in MTBD, but weakens the interaction in VCR and in NTD. The additional phosphorylation at Ser184 and Thr220 results in stronger binding of d14‐3‐3ζ and m14‐3‐3ζ in the vicinity of the phosphorylated residues and weaker binding elsewhere, with the exception of CTD that strongly binds m14‐3‐3ζ.

### Phosphorylated MAP2c residues upon 14‐3‐3ζ binding show an exchange contribution to line broadening

The NMR data imply that multiple binding sites at MAP2c contribute to the interaction between MP‐MAP2c and 14‐3‐3ζ, whereas ITC shows only an overall view of the binding affinities of the different binding sites. As NMR allows us to distinguish most of the individual phosphorylated residues, we attempted to determine the affinity of the phosphorylated residues for d14‐3‐3ζ by monitoring the peak broadening in the HNCO spectra. However, the heights of the peaks of the phosphorylated residues, pSer184, pThr220, and pSer435, at a concentration of 14‐3‐3ζ eight times lower decreases to less than half in HNCO spectra (Fig. 2C,D). The decrease of the heights of the phosphorylated peaks in the presence of d14‐3‐3ζ is different in HNCO and ^1^H‐^15^N HSQC spectra, which differ by the pulse sequences (Fig. 2F). It suggests that an exchange contributes to the peak broadening (Fig. 1) and that the HNCO and HSQC peak heights cannot be used to determine the affinity of the phosphorylated residues.

### Monitoring of 14‐3‐3ζ binding by ^31^P NMR spectroscopy

**Fig. 3:**
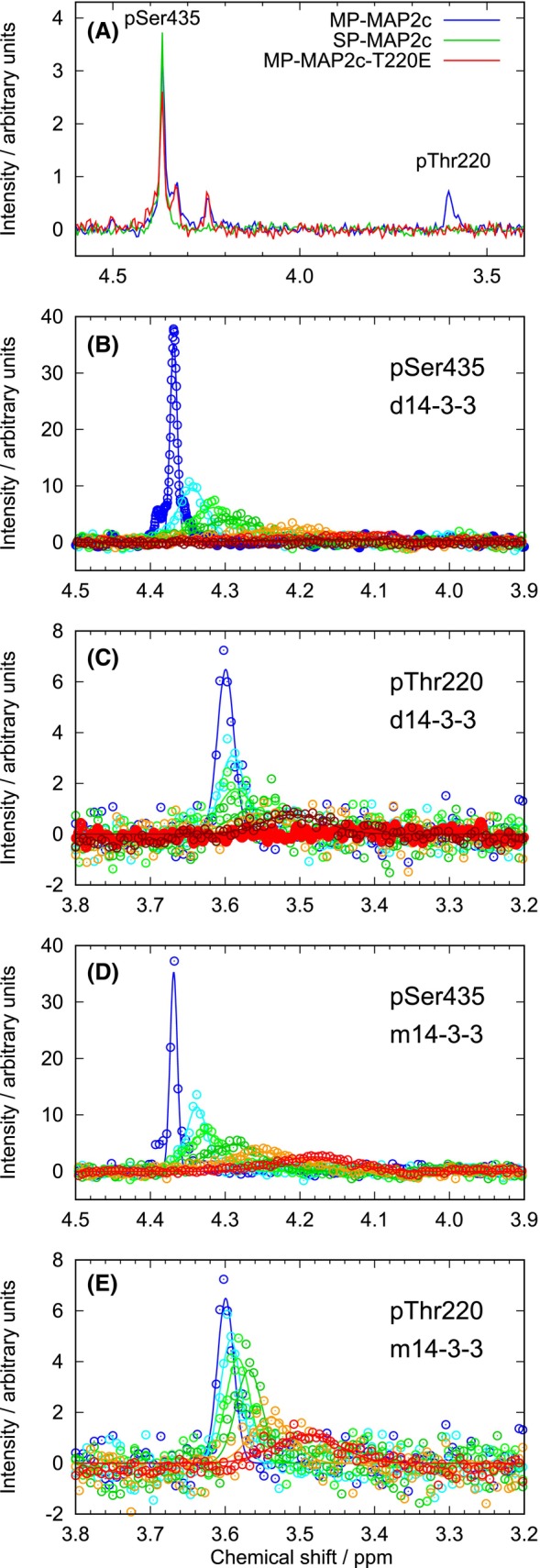
^31^P NMR spectra used for the assignment of peaks of MAP2c phosphorylated by cAMP‐dependent protein kinase and monitoring of the dimeric 14‐3‐3ζ (d14‐3‐3ζ) and monomeric 14‐3‐3ζ (m14‐3‐3ζ) binding. (A) ^31^P NMR spectra of MAP2c fully phosphorylated by PKA (MP‐MAP2c, blue), MAP2c selectively phosphorylated by PKA at Ser435 (SP‐MAP2c, green), and PKA phosphorylated MAP2c‐T220E (red). (B–E) Changes of the ^31^P peak of pSer435 in SP‐MAP2c (B, D) and pThr220 in MP‐MAP2c (C, E) upon addition of 12.5 μm (light blue), 25 μm (light green), 50 μm (green), 100 μm (orange), 200 μm (red), and 300 μm (brown) d14‐3‐3ζ (B, C, monomeric concentrations) and m14‐3‐3ζ (D, E). Circles in Panels B–E represent the actual data points; the curves were fitted to the data in order to determine the positions of peak maxima. The reproducibility of the binding of 14‐3‐3ζ was confirmed by three independent titrations.

**Fig. 4:**
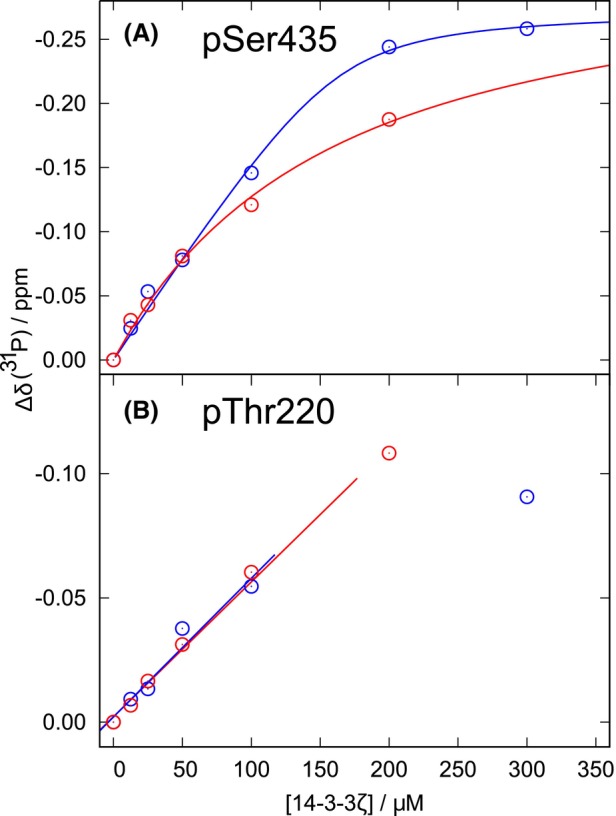
^31^P chemical shifts of phosphorylated serines upon addition of 14‐3‐3ζ. Changes of ^31^P chemical shifts of pSer435 in MAP2c selectively phosphorylated by cAMP‐dependent protein kinase (PKA) at Ser435 (SP‐MAP2c, A) and of pThr220 (B) in MAP2c fully phosphorylated by PKA (MP‐MAP2c) upon titration by dimeric14‐3‐3ζ (d14‐3‐3ζ, blue circles) and monomeric 14‐3‐3ζ (m14‐3‐3ζ, red circles). The curves plotted in Panel A, blue for d14‐3‐3ζ, red for m14‐3‐3ζ, were obtained by fitting the data to the equation $\Delta \delta =A\left(x+\mathrm{nc}+K_{D}-\sqrt{{\left(x+\mathrm{nc}+K_{D}\right)}^{2}-4\mathrm{ncx}}\right)/2n$, where *A* is a coefficient, *c* is the MAP2c concentration, *x* is concentration of 14‐3‐3ζ monomers, *K*_D_ is the apparent dissociation constant, and *n* is the stoichiometry coefficient. The lines plotted in Panel B (blue for d14‐3‐3ζ, red for m14‐3‐3ζ) were obtained by fitting the data to the linear equation ∆δ = *a* + *bx*, where *x* is the concentration of 14‐3‐3ζ monomers and *a*, *b* are the coefficients. The plotted data were calculated from spectra presented in Fig. 3.

To obtain a further insight, we monitored changes of the ^31^P peaks upon titration of 60 μm SP‐MAP2c and MP‐MAP2c with d14‐3‐3ζ and m14‐3‐3ζ. The ^31^P 1D NMR spectrum of MP‐MAP2c showed an isolated peak at 3.61 p.p.m. and a group of partially overlaid peaks in the region between 4.2 and 4.5 p.p.m. Using SP‐MAP2c and the MP‐MAP2c mutants T220E and S435D, we assigned the peak at 3.61 p.p.m. to pThr220 and found that pSer184 and pSer435 contribute to the peak at 4.37 and at 4.33 p.p.m. (Fig. 3A). Upon addition of 14‐3‐3ζ, the ^31^P peaks shifted and broadened (Fig. 3B–E), indicating an intermediate‐to‐fast exchange regime. In agreement with the HNCO experiment, peak heights decreased substantially even at the lowest (12.5 μm) 14‐3‐3ζ concentrations. The peak broadening did not allow us to analyze phosphoserines of MP‐MAP2c quantitatively. Therefore, we present data only for pSer435 of SP‐MAP2c and well‐resolved pT220 of MP‐MAP2c (Fig. 4). The peak areas remained roughly constant, as expected.

The chemical shifts of pThr220 in MP‐MAP2c could be interpreted only qualitatively, as pThr220 is only one of several competing binding sites with non‐identical affinities. The chemical shift changed approximately linearly at low d14‐3‐3ζ (blue line in Fig. 4B) and m14‐3‐3ζ (red line in Fig. 4B) concentrations. The slope of the change was similar for d14‐3‐3ζ and m14‐3‐3ζ. In the case of the MP‐MAP2c:d14‐3‐3ζ complex, the line broadening did not allow us to determine the chemical shift at 200 μm concentration of d14‐3‐3ζ. Changes in the chemical shifts were also detected beyond the 1 : 1 MAP2c:m14‐3‐3ζ ratio observed by ITC, which may be explained by the m14‐3‐3ζ dimerization at higher concentrations.

A more quantitative interpretation was possible for the chemical shift changes of pSer435 in SP‐MAP2c. Fitting of chemical shift changes during titration by d14‐3‐3ζ (blue curve in Fig. 4A) provided maximum chemical shift change of (−0.27 ± 0.02) p.p.m. and *n* = 2.73 ± 0.20. The binding was too strong to determine *K*_D_ reliably (the fitted value of (7.3 ± 7.8) μm suggests *K*_D_ lower than 15 μm). A weaker binding of unphosphorylated SP‐MAP2c sites thus seems to influence the overall *K*_D_ = (74 ± 21) μm obtained by ITC more than the interaction of pSer435. Fitting of chemical shift changes during titration by m14‐3‐3ζ (red curve in Fig. 4A) provided a maximum chemical shift change of (−0.32 ± 0.3) p.p.m. and a relatively well‐defined *K*_D_ = (133 ± 28) μm. Stoichiometry could not be determined for such high *K*_D_ at the 60 μm SP‐MAP2c concentration used in the ^31^P experiment. The *K*_D_ is comparable to that determined by ITC. However, the values obtained in the ^31^P experiment may be biased by m14‐3‐3ζ dimerization at higher concentrations.

In conclusion, changes of the ^31^P chemical shift upon d14‐3‐3ζ and m14‐3‐3ζ titration are consistent with the HNCO peak broadening presented in Fig. 1 and complement the ITC results. The gradual chemical shift change is indicative for an intermediate‐to‐fast binding process, expected for the observed *K*_D_ values.

### Oligomeric states of MP‐MAP2c complexes with d14‐3‐3ζ and m14‐3‐3ζ

**Fig. 5:**
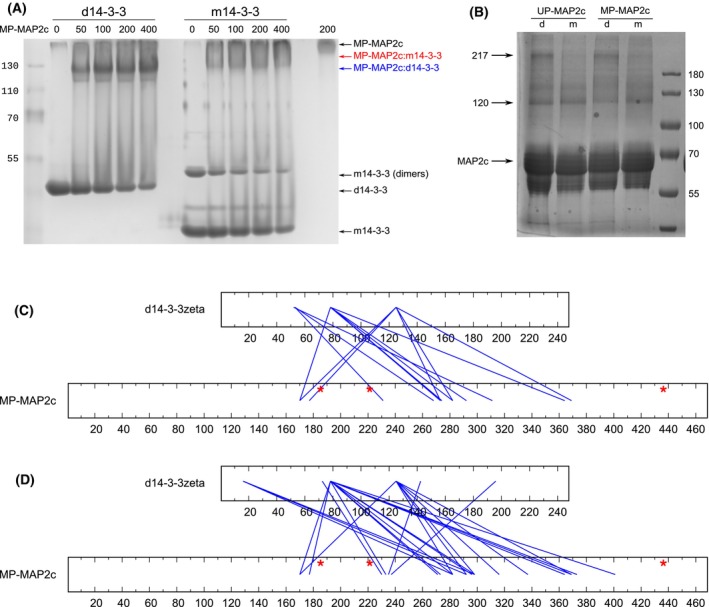
Determination of oligomeric states of MAP2c fully phosphorylated by cAMP‐dependent protein kinase (MP‐MAP2c) complexes with d14‐3‐3ζ and m14‐3‐3ζ using native PAGE and chemical crosslinking. (A) Native PAGE of MP‐MAP2c with dimeric14‐3‐3ζ (d14‐3‐3ζ) and monomeric 14‐3‐3ζ (m14‐3‐3ζ). The concentration of d14‐3‐3ζ and m14‐3‐3ζ was 100 μm, and the concentration of MP‐MAP2c varied from 0 to 400 μm. The composition of samples is indicated above the gel. MP‐MAP2c:d14‐3‐3 and MP‐MAP2c:m14‐3‐3 label the complex of MP‐MAP2c with d14‐3‐3ζ and m14‐3‐3ζ, respectively, and m14‐3‐3 (dimers) labels the portion of m14‐3‐3ζ present in dimers. The results presented have been obtained as duplicates. (B) SDS/PAGE of cross‐linked complexes of unphosphorylated MAP2c (UP‐MAP2c) and MP‐MAP2c with d14‐3‐3ζ (d) and m14‐3‐3ζ (m). The following samples were loaded to the gel: UP‐MAP2c + d14‐3‐3ζ, UP‐MAP2c + m14‐3‐3ζ, MP‐MAP2c + d14‐3‐3ζ, MP‐MAP2c + m14‐3‐3ζ. The complexes formed between UP‐MAP2c/MP‐MAP2c and d14‐3‐3ζ/m14‐3‐3ζ are indicated by arrows at 120 and 217 kDa, respectively. Crosslinking between UP‐MAP2c/MP‐MAP2c with d14‐3‐3ζ/m14‐3‐3ζ has been replicated in an independent experiment. (C, D) Residues of MP‐MAP2c binding to d14‐3‐3ζ determined by crosslinking and mass spectrometry. Results of the analysis of the bands migrating as 120 kDa (C) and 217 kDa (D) globular proteins on the gel shown in Panel B are presented. The red asterisks represent the phosphorylation sites.

**Fig. 6:**
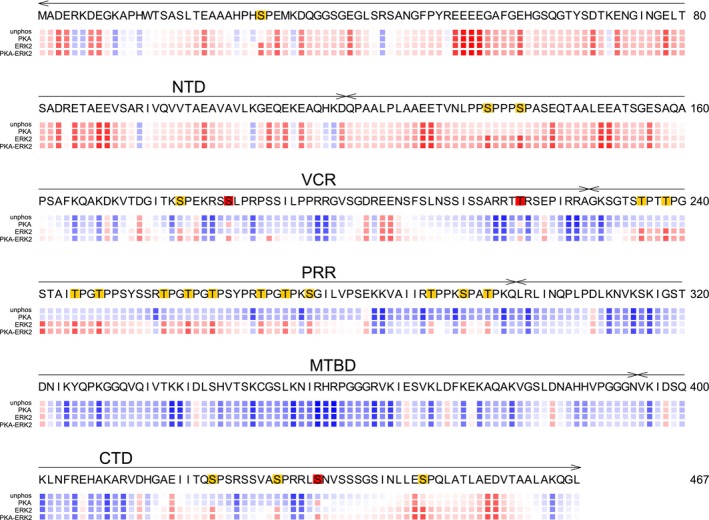
Electrostatic potential of unphosphorylated and phosphorylated MAP2c. The relative electrostatic potential is shown as color‐coded boxes below the sequences for unphosphorylated (upper row, labeled “unphos”) and phosphorylated (lower rows with the kinase indicated) MAP2c. The potential is approximated by ∑_*j*_ *CQ*_*i*_/(*d*_0_ + *d*_1_|*n*_*i*_ − *n*_*j*_|), where *Q*_*i*_ and *n*_*j*_ are charge and sequential number of the *i*‐th residue, *C* is a constant including the electric permittivity, and *d*_*k*_ are distance constants [21]. The ratio *d*_1_/*d*_0_ was set to 2.0 and the colors were chosen so that red and blue correspond to the highest negative and positive potential, respectively, which makes the color code independent of *C*/*d*_0_. The residues phosphorylated by cAMP‐dependent protein kinase (PKA) and extracellular signal‐regulated kinase 2 (ERK2) are highlighted in red and yellow, respectively.

We used native polyacrylamide gel electrophoresis (PAGE) to monitor the size of MP‐MAP2c in complex with d14‐3‐3ζ (Fig. 5A). As p*I* of MP‐MAP2c (~ 8.6, cf. Fig. 6) is close to pH of the running buffer (~ 8.4), MP‐MAP2c migrates slowly in native PAGE and stays at the top of the gel. In the presence of d14‐3‐3ζ, a band migrating as the ~ 130 kDa standard appeared on the gel, in addition to the d14‐3‐3ζ band. In the presence of m14‐3‐3ζ, a band migrating slower than that of the complex with d14‐3‐3ζ was observed, suggesting a less negatively charged complex of MP‐MAP2c:d14‐3‐3ζ than for MP‐MAP2c:m14‐3‐3ζ. In addition, native PAGE showed that approximately 20% of m14‐3‐3ζ is present as a dimer at 100 μm.

We employed chemical crosslinking with disuccinimidyl dibutyric urea (DSBU) which crosslinks lysine residues, to confirm the formation of the complexes between MP‐MAP2c and 14‐3‐3ζ (Fig. 5B). In sodium dodecyl sulfate polyacrylamide gel electrophoresis (SDS/PAGE), UP‐MAP2c and MP‐MAP2c migrate at ~ 70 kDa, as seen previously [56], while the dimer d14‐3‐3ζ migrates at ~ 56 kDa. Using 20 μm UP‐MAP2c or MP‐MAP2c and 80 μm d14‐3‐3ζ or m14‐3‐3ζ, we observed a band at ~ 120 kDa, corresponding to a 1 : 2 (expressed as d14‐3‐3ζ monomer) complex UP‐MAP2c/MP‐MAP2c:d14‐3‐3ζ/m14‐3‐3ζ. We also observed a slower band for d14‐3‐3ζ, but not for m14‐3‐3ζ, in the presence of UP‐MAP2c or MP‐MAP2c. The slower band migrates as 217 kDa globular proteins, which corresponds to a 2 : 2 complex. Mass spectrometry confirmed the presence of the intermolecular cross‐links in the described bands for MP‐MAP2c:d14‐3‐3ζ (Table 2 and Fig. 5C,D). The cross‐links were formed close to pSer184 and pThr220, and in the MTBD of MAP2c. MAP2c does not contain lysines close to pSer435, explaining why no cross‐links are observed in this region. Taken together, these results suggest that each site of UP‐MAP2c or MP‐MAP2c binds one dimer of d14‐3‐3ζ, while each phosphorylated site of MP‐MAP2c would bind one monomer of m14‐3‐3ζ independently, making the complex MP‐MAP2c:d14‐3‐3ζ more compact than m14‐3‐3ζ. In addition, the remaining free binding sites on UP‐MAP2c and MP‐MAP2c are able to bind one additional dimer of d14‐3‐3ζ, reflected by the additional cross‐links between the VCR and MTBD of MAP2c and d14‐3‐3ζ.

**Table 2:** Residues of MP‐MAP2c and d14‐3‐3ζ involved in crosslinking determined using mass spectrometry. The bands at 120 and 217 kDa are the bands of the gel shown in Fig. 5. The score reflects the similarity of the experimental MS/MS data with theoretical MS/MS data (*m/z* values calculated from expected fragmentation mechanisms).

Residues		Score	
d14‐3‐3ζ	MP‐MAP2c	Band 217 kDa	Band 120 kDa
K14	K282	–	54
K14	K292	–	102
K14	T296	–	148
T72	K230	–	162
Y51	K311	5‐‐	–
K52	S231	140	‐‐
K52	T268	52	–
K77	K273	121	–
K77	S274	–	70
K77	K292	101	–
K78	K170	75	134
K78	S178	–	95
K78	T271	–	95
K78	S274	111	–
K78	S280	92	82
K78	K282	57	85
K78	K292	69	–
K78	T296	–	121
K78	K298	–	168
K78	K364	171	53
K78	K369	–	116
K78	K373	74	143
K78	K401	–	54
K123	K170	182	171
K123	T173	63	–
K141	T235	–	150
K196	T237	–	54
K123	K273	144	–
K123	S280	–	174
K123	K282	181	71
K123	S293	106	147
K123	K298	–	49
K123	K337	–	49
K123	S315	–	47
K123	K369	162	186

### Structural details of phosphorylated and unphosphorylated peptides bound to d14‐3‐3ζ

**Fig. 7:**
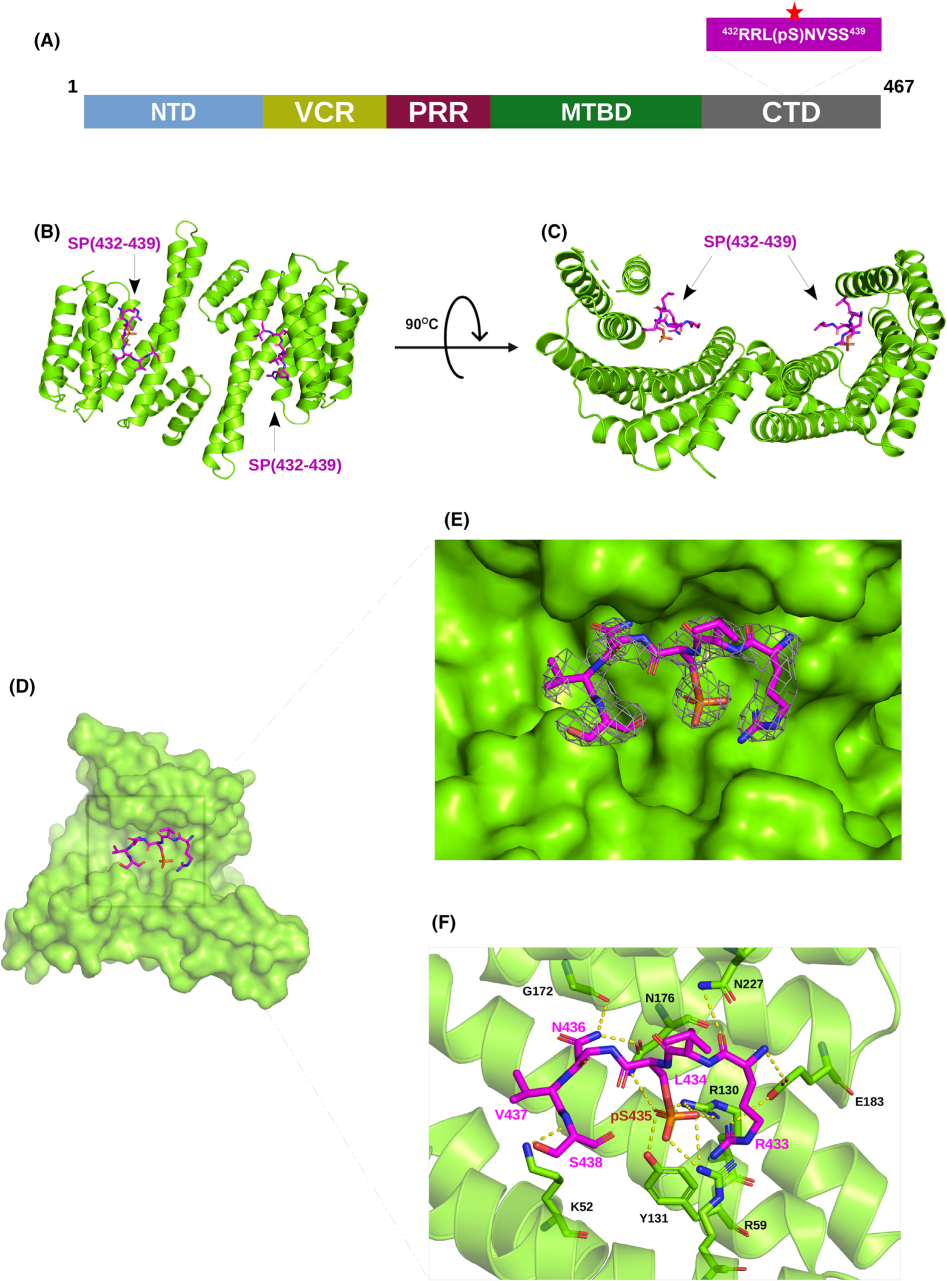
Crystal structure of the phosphopeptide corresponding to residues 432–439 of MAP2c (SP‐(432–439)) in complex with dimeric 14‐3‐3ζ (d14‐3‐3ζ). (A) Schematic diagram of the MAP2c domains along with the peptide sequence. The red star indicates the phosphorylated residue. (B, C) View of the dimeric complex of d14‐3‐3ζ (green, cartoon representation) and the phosphopeptide SP‐(432–439) (magenta, cartoon representation). (D) View of the monomer in the dimeric complex of d14‐3‐3ζ (green, surface representation) and SP‐(432–439) (magenta, stick representation). (E) 2*F*_o_ − *F*_c_ electron density map contoured at 1.0σ for SP‐(432–439) (magenta). (F) Detailed view of the binding groove of SP‐(432–439). SP‐(432–439) is shown in magenta as stick representation with the phosphorus atom shown in orange. The peptide shows the typical elongated conformation when bound to d14‐3‐3ζ (in cartoon representation in green). The essential amino acids (in stick representation) of the d14‐3‐3ζ protein involved in the interaction are labeled. Dotted lines indicate polar contacts. The images were created using the pymol Molecular Graphic System (Schrödinger LLC, version 2.2.3).

**Fig. 8:**
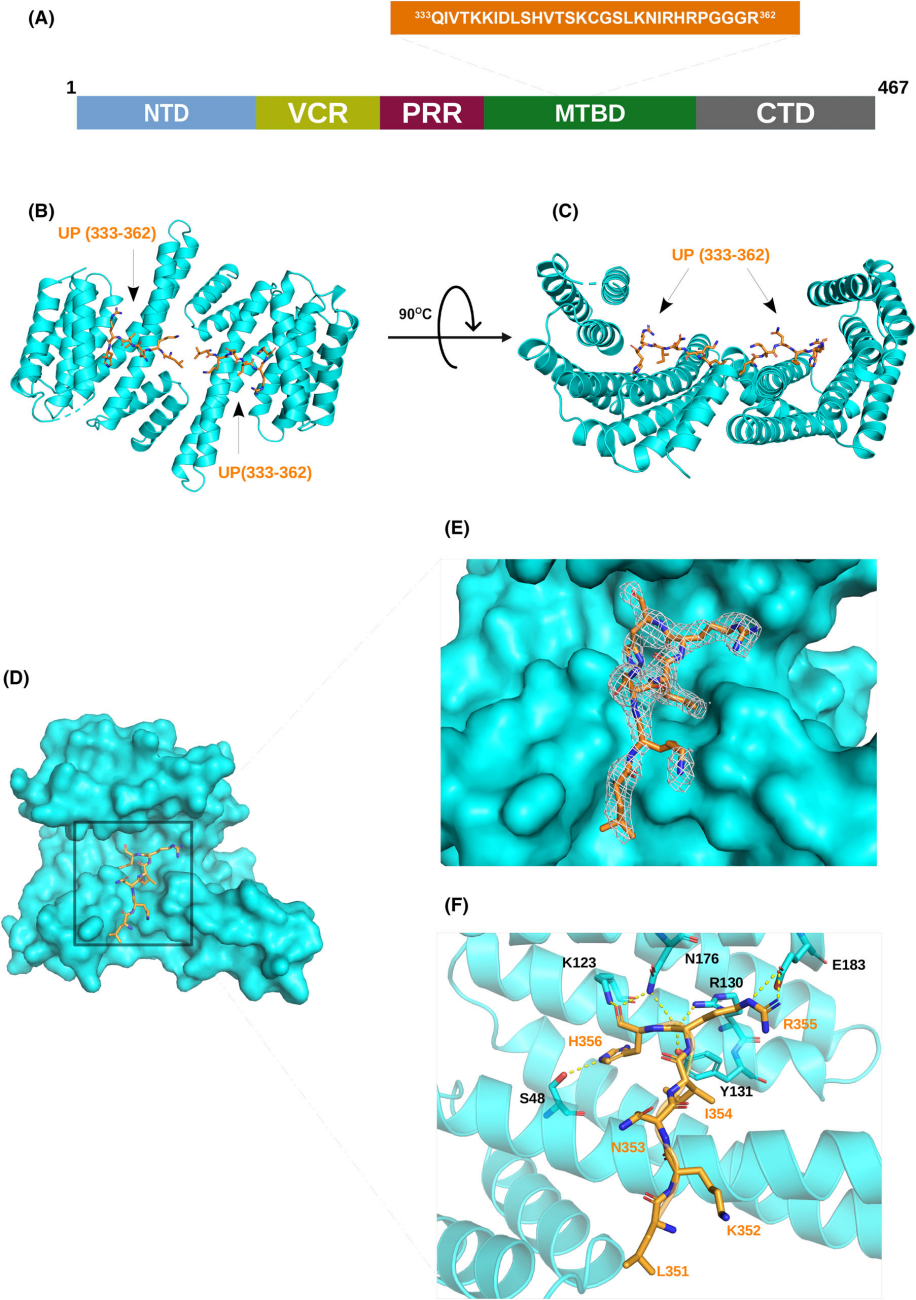
Crystal structure of the peptide corresponding to residues 432–439 of MAP2c (UP‐(333–362)) in complex with dimeric 14‐3‐3ζ (d14‐3‐3ζ). (A) Schematic diagram of the MAP2c domain along with the peptide sequence. (B, C) View of the dimeric complex of d14‐3‐3ζ (cyan, cartoon representation) and the UP‐(333–362) (orange, cartoon representation). (D) View of the dimeric complex of d14‐3‐3ζ (cyan, surface representation) and the peptide UP‐(333–362) (orange, stick representation). (E) 2*F*_o_ − *F*_c_ electron density map contoured at 1.0σ for UP‐(333–362) (orange). (F) Detailed view of UP‐(333–362) (in orange as stick representation) bound to d14‐3‐3ζ (in cartoon representation in cyan). The essential amino acids of the d14‐3‐3ζ protein involved in the interaction are labeled. Dotted lines indicate polar contacts. The essential amino acids of d14‐3‐3ζ protein involved in the interaction are labeled. Dotted lines indicate polar contacts. The images were created using the pymol Molecular Graphic System (Schrödinger LLC, version 2.2.3).

**Fig. 9:**
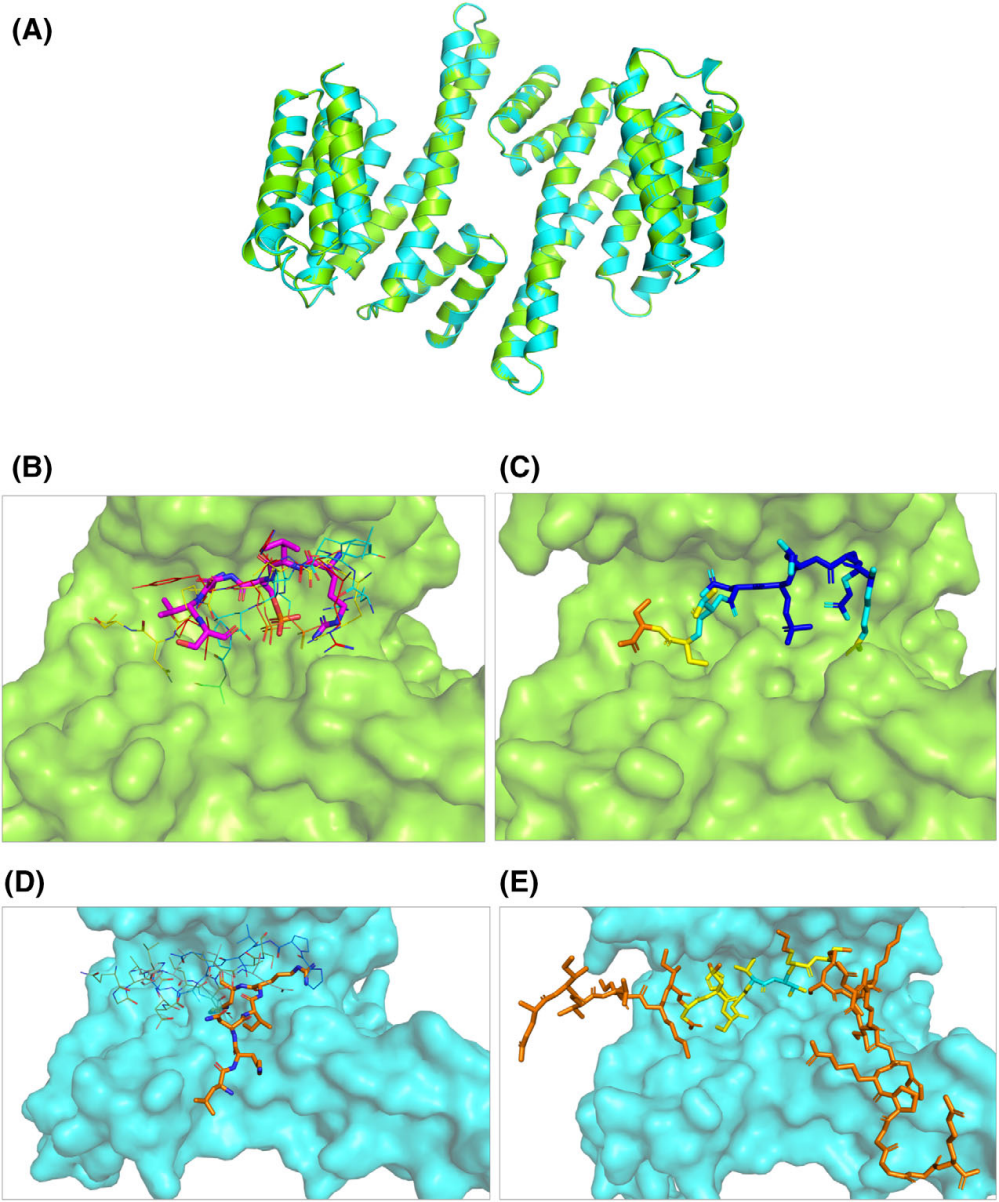
Structural comparison of the dimeric 14‐3‐3ζ (d14‐3‐3ζ) binding interface. (A) Structural alignment of d14‐3‐3ζ with phosphopeptide corresponding to residues 432–439 of MAP2c (SP‐(432–439), green, ribbon representation) and d14‐3‐3ζ with peptide corresponding to residues 333–362 of MAP2c (UP‐(333–362), cyan, ribbon representation). (B) Surface representation of the d14‐3‐3ζ (green) pocket accommodating pSer435 of MAP2c peptide SP‐(432–439), shown in magenta as a stick model. Superimposed are other structurally elucidated d14‐3‐3ζ binding phosphoresidue recognition motifs as line models: cRAF (PDB ID: 3NKX, in yellow [57]); nuclear export signal phosphopeptide (PDB ID: 1QJB, in red [28]); and peptide sequence RLYH(pS)LPA (PDB ID: 1QJA, in cyan [28]). (C) AlphaFold 3 prediction of the d14‐3‐3ζ (green, surface) pocket accommodating the phosphopeptide SP‐(432–439), shown in stick representation and colored according to the predicted local distance difference test (pLDDT) score: blue, pLDDT > 90; cyan, 90 > pLDDT > 70; yellow, 70 > pLDDT > 50; orange pLDDT < 50. (D) Surface representation of the d14‐3‐3ζ (blue) pocket accommodating unphosphorylated MAP2c peptide UP‐(333–362) (shown in orange as a stick model). Superimposed are other structurally elucidated d14‐3‐3ζ binding motifs for unphosphorylated targets as line models: ExoS peptide (PDB ID: 4N7G, in green [66]), R18 peptide (PDB ID: 1A38, in white [65]), and the glycosylated Ser‐O‐GlcNAc peptide (PDB ID: 6BYJ, in blue [67]). (E) AlphaFold 3 prediction of the d14‐3‐3ζ (cyan, surface) pocket accommodating the peptide UP‐(333–362), shown in stick representation and colored as in Panel C. The images were created using the pymol Molecular Graphic System (Schrödinger LLC, version 2.2.3).

In order to study the structural details of the interaction of MAP2c with d14‐3‐3ζ, we designed two MAP2c peptides, based on the NMR binding studies: a peptide of the sequence RRL(pS)NVSS, corresponding to MAP2c residues 432–439 and spanning the phosphorylated binding site pSer435, referred to as SP‐(432–439) below; and a peptide of the sequence QIVTKKIDLSHVTSKCGSLKNIRHRPGGGR, corresponding to MAP2c residues 333–362 and spanning the main interaction site in MTBD of UP‐MAP2c, referred to as UP‐(333–362) below. ITC showed that the peptides bind with *K*_D_ of (3.2 ± 0.4) mm and (1.8 ± 0.1) mm, respectively (Fig. S9). We solved the crystal structures of d14‐3‐3ζ in complex with peptides SP‐(432–439) (Fig. 7, PDB ID: 9FUM) and UP‐(333–362) (Fig. 8, PDB ID: 9FVL) to resolutions of 2.45 and 2.08 Å, respectively (Table 3). For the d14‐3‐3ζ protein, we found interpretable electron density for 225 and 226 residues respectively, out of the 230 residues examined. The 14‐3‐3ζ protein adopts a dimeric conformation, with each monomer comprising nine α‐helices arranged in a characteristic cup‐like shape featuring a highly conserved amphipathic groove essential for binding phosphorylated peptides. The dimer interface is stabilized by a network of hydrogen bonds and hydrophobic interactions, with key residues such as Ala 19, Arg 21, Asp24, Ser61, Lys77, Tyr85, Lys88, and Glu92 involved in maintaining the structural integrity of the dimer. The individual monomeric chains of d14‐3‐3ζ in the structures of complexes with SP‐(432–439) and UP‐(333–362) exhibited a root mean square deviation (RMSD) of 0.239 Å (over 175 Cα atoms) and 0.181 Å (over 164 Cα atoms), respectively, indicating minimal variation between the individual chains within each structure. Furthermore, the overall comparison between the two solved d14‐3‐3ζ structures shows a highly conserved fold, with an RMSD of 0.151 Å over 416 Cα atoms, suggesting a structural consistency of 14‐3‐3ζ across both complexes (Fig. 9A).

**Table 3:** Data collection and refinement statistics of the crystals of d14‐3‐3ζ and MAP2c peptides.

	d14‐3‐3ζ:SP‐(432–439)	14‐3‐3ζ:UP‐(333–362)
Space group	P 1 21 1	P 1 21 1
X‐ray source	PETRA III, EMBL c/o DESY BEAMLINE P14 (MX2)	PETRA III, EMBL c/o DESY BEAMLINE P14 (MX2)
Wavelength (Å)	0.9762	0.9762
Data collection temperature (K)	100	100
Cell dimensions		
*a*, *b*, *c* (Å)	74.39, 69.14, 82.80	74.31, 69.11, 82.62
α, β, γ (°)	90.00, 112.00, 90.00	90.00, 112.09, 90.00
Resolution (Å)	48.83–2.45	48.78–2.08
Total number of reflections	222 336 (24 391)	325 384 (24 898)
Number of unique reflections	28 553 (3151)	46 317 (3557)
Completeness (%)	99.1 (97.9)	99.3 (98.6)
Mean *I*/σ (*I*)	11.8 (1.9)	11.7 (2.0)
Rmerge (%)	0.128 (1.264)	0.102 (0.977)
Rpim (%)	0.049 (0.479)	0.042 (0.395)
Rmeas (%)	0.137 (1.354)	0.111 (1.055)
CC1/2 (%)	0.999 (0.690)	0.998 (0.805)
Multiplicity	7.8	7.0
Refinement	REFMAC 5.8.0425	REFMAC 5.8.0425
Rwork/Rfree (%)	21.0/27.5	23.56/28.20
R.M.S. deviations		
Bond lengths (Å)	0.008	0.0082
Bong angles (Å)	1.9189	1.8955
Number of molecules in AU	3	3
Solvent content (%)	49.59	47.39
Number of non‐H protein atoms	3903	3987
Number of water molecules	153	222
Wilson *B*‐factor (Å^2^)	42	31.2
Average *B*‐factor (Å^2^)	52.816	39.0
Ramachandran analyses		
Favored (%)	100	99.7
Disallowed (%)	0	0.3

For the d14‐3‐3ζ:SP‐(432–439) structure, the final density allowed the building of 4 and 6 out of 8 residues on the individual monomeric chains. The overall structure aligns well with previously reported 14‐3‐3 isoforms binding diverse phosphorylated client proteins such as cRAF, nuclear export signal phosphopeptide, and the RLYH(pS)LPA peptide [28, 57] (Fig. 9B). The d14‐3‐3ζ displays a central binding groove that accommodates the phosphorylated 14‐3‐3 interaction motifs of MAP2c (Fig. 7A–E). The phosphate moiety of pSer435 forms electrostatic interactions with the positively charged pocket consisting of Arg59, Arg130, and Tyr131 (Fig. 7D–F). Additional main‐chain contacts of SP‐(432–439) are formed with Gly172, Asn176, Glu183, and Asn227 from d14‐3‐3ζ. The side‐chain interactions are observed for MAP2c‐Asn436, interacting with Gly172 and Asn176 of d14‐3‐3ζ, and for MAP2c‐Ser435, interacting with the main chain of d14‐3‐3ζ‐Arg59.

**Fig. 10:**
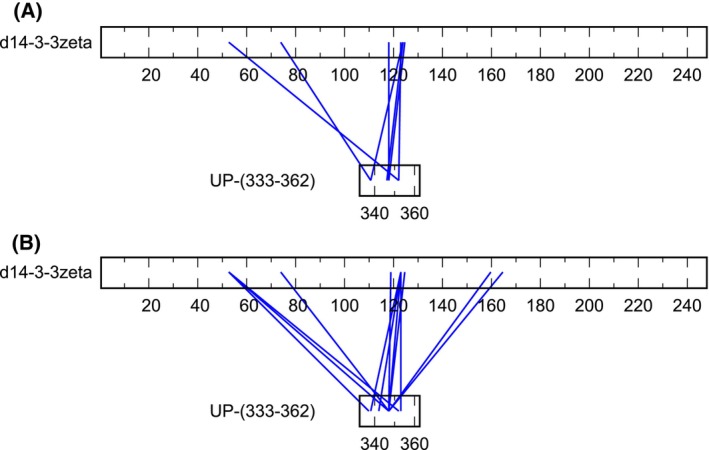
Residues of peptide corresponding to residues 333–362 of MAP2c (UP‐(333–362)) binding to dimeric 14‐3‐3ζ (d14‐3‐3ζ) determined by crosslinking. Mass spectrometry results of the crosslinking reactions of (A) 80 μm d14‐3‐3ζ and 100 μm UP‐(333–362) and (B) 80 μm d14‐3‐3ζ and 400 μm UP‐(333–362).

For the d14‐3‐3ζ:UP‐(333–362) complex, we found interpretable density for 6 residues out of 29 residues. The identification of the binding region in this case was more challenging. The observed electron density clearly corresponding to an isoleucine side chain was used for building the peptide chain. The binding region of the peptide was consistent with the electron density of residues _351_LKNIRH_356_ of MAP2c and with the positions of possible entry sites of UP‐(333–362) regions not observed in the crystal. The contacts are in a good agreement with MAP2c MTBD domain:d14‐3‐3ζ interactions observed as peak broadening in HNCO spectra (Figs 1 and 2A,E) and consistent with the crosslinking data (Fig. 10 and Table 4). The binding mode suggests that the ligand binding site on d14‐3‐3ζ consisting of Lys123, Arg130, Tyr131, and Glu183 makes ionic and hydrogen bonding interactions with Arg355 and His356 residues on the MAP2c. Asn53 makes main chain contacts with UP‐(333–362) (Fig. 8D–F). A closer look at the complex with UP‐(333–362) suggests that the unphosphorylated MAP2c‐derived UP‐(333–362) occupies a non‐canonical binding pocket on d14‐3‐3ζ.

**Table 4:** Residues of UP‐(333–362) and d14‐3‐3ζ involved in crosslinking determined using mass spectrometry. The concentration of d14‐3‐3ζ:UP‐(333–362) mixture (in μm) was 80 : 100 (Sample 1) and 80 : 400 (Sample 2). Residues of UP‐(333–362) are numbered according to MAP2c sequence. The score reflects the similarity of the experimental MS/MS data with theoretical MS/MS data (*m/z* values calculated from expected fragmentation mechanisms).

Residues		Score	
d14‐3‐3ζ	UP‐(333–362)	Sample 1	Sample 2
K52	T336/K337	–	104
K52	K352	118	125
K52	T345	–	138
K52	T345/S346/K347	–	112
K78	T336/K337/K338	126	–
K78	S346/K347	–	213
K117/K118	T345/S346/K347	190	208
K123	S342	–	214
K123	K338	–	120
K123	K352	164	177
K123/K125	K338	159	–
K123/K125	T345/S346/K347	174	150
K125	T345/S346/K347	115	130
K160/K161	S346/K347	–	102
T166	K347	–	110

A comparative analysis with AlphaFold 3 [58] predictions of d14‐3‐3ζ bound to SP‐(432–439), which contains a post‐translational modification at Ser435, revealed that the experimental and predicted structures were structurally similar, with the predicted model showing high confidence for peptide residues (Fig. 9C). In contrast, the AlphaFold‐predicted structure of UP‐(333–362) exhibited high confidence (predicted local distance difference test score between 70 and 90) for only two residues, and did not resemble the experimental structure (Fig. 9E). While the predicted structure partially overlapped with the peptide, it predominantly occupied the canonical binding pocket, in a stark contrast to our experimental results. It indicates that AlphaFold 3 reliably predicts structures of 14‐3‐3 complexes with phosphopeptides in the canonical binding pocket, but predictions of complexes with unphosphorylated disordered peptides are challenging.

### ERK2 phosphorylation interferes with the binding of UP‐MAP2c and MP‐MAP2c to 14‐3‐3ζ

We have shown previously that MAP2c is phosphorylated in the neuroblastoma cell extract by PKA, but also by proline‐directed kinases such as ERK2 [13]. It is therefore probable that MAP2c in brain would be phosphorylated by a combination of PKA and proline‐directed kinases. We previously determined the phosphorylation sites of the proline‐directed kinase ERK2, highly expressed in the brain, on MAP2c [13]. Most of the phosphorylation sites are located in PRR, but three sites (pSer178, pSer430, and pSer448) are close to the PKA phosphorylation sites. Additional phosphorylation sites in proximity to PKA phosphorylated Tau (pTau) binding sites reduce binding affinity to 14‐3‐3ζ [55]. We speculated that these additional phosphorylation sites could interfere with 14‐3‐3ζ binding. We therefore checked how ERK2 phosphorylation of MAP2c influences the interaction with d14‐3‐3ζ and m14‐3‐3ζ.

We recorded 3D HNCO spectra of MAP2c phosphorylated by ERK2 and MAP2c phosphorylated by PKA followed by ERK2. The results are shown in Fig. 1D,E. As expected from the consensus 14‐3‐3ζ binding motifs, in the presence of d14‐3‐3ζ and m14‐3‐3ζ, the peak heights of the ERK2‐phosphorylated residues do not decrease, except for pSer274 in a middle of a region heavily phosphorylated by ERK2, and the peak broadening in the VCR and MTBD is less pronounced than in UP‐MAP2c. ERK2 and other proline‐directed Ser/Thr protein kinases do not create good 14‐3‐3 binding sites in general [59]. Interestingly, Ser274 in the sequence _270_GTPKpSGILV_278_ is the only residue of MAP2c not followed by proline, but phosphorylated by ERK2.

On the other hand, when MAP2c is phosphorylated by PKA followed by ERK2, the peak heights of d14‐3‐3ζ decrease more in the VCR, next to pSer184 and pThr220, and in the MTBD, while the peak heights of the residues in the CTD decrease less in the presence of both d14‐3‐3ζ and m14‐3‐3ζ. Our results show that residues phosphorylated by proline‐directed kinases are much worse targets for 14‐3‐3ζ binding than those phosphorylated by PKA. ERK2 phosphorylation interferes with the binding of unphosphorylated residues of MAP2c as well as phosphorylated pSer435 to d14‐3‐3ζ.

In presence of d14‐3‐3ζ, the HNCO peak heights decrease also in the regions 40–60 and 90–134, showing that phosphorylation by PKA and ERK2 reinforces the interaction between the NTD and CTD of MAP2c seen in UP‐MAP2c.

## Discussion

We characterized interactions of multiple binding sites of a microtubule‐associated protein MAP2c with a regulatory protein 14‐3‐3ζ. MAP2c binds to the major dimeric form d14‐3‐3ζ via both phosphorylated and unphosphorylated residues. Unphosphorylated MAP2c binds to d14‐3‐3ζ through its MTBD, Ser435, and a few sites in VCR, while the C‐terminus does not bind d14‐3‐3ζ. Phosphorylation of Ser435 in CTD and of Ser184 and Thr220 in VCR makes these residues better ligands of d14‐3‐3ζ, which is documented by a somewhat lower overall *K*_D_ and weaker interactions of d14‐3‐3ζ with other regions of MAP2c, most notably with MTBD. On the other hand, it should be stressed that Ser184, Thr220, and Ser435 (a) are not present in the ideal canonical mode I or mode II phosphopeptide sequences [26, 27], and (b) define MAP2c regions that bind d14‐3‐3ζ also without phosphorylation.

The overall *K*_D_ values of d14‐3‐3ζ complexes with both UP‐MAP2c and MP‐MAP2c are comparable to the expected 14‐3‐3ζ concentrations in the brain [60, 61, 62, 63], supporting the regulatory role of the interactions. However, the measured values represent an average for interactions of multiple MAP2c regions with presumably two binding sites of d14‐3‐3ζ. Therefore, experiments with selectively phosphorylated MAP2c at Ser435 (SP‐MAP2c) provided a useful insight, showing that d14‐3‐3ζ can bind phosphorylated and unphosphorylated residues simultaneously.

In order to learn if unphosphorylated MTBD interacts with the same binding pocket of d14‐3‐3ζ as the phosphorylated residues, we solved crystal structures of d14‐3‐3ζ complexes with peptides UP‐(333–362), corresponding to MTBD, and SP‐(432–439), corresponding to the site containing pSer435. The crystal structures of d14‐3‐3ζ with MAP2c peptides show that the unphosphorylated and phosphorylated peptide bind two overlapping but distinct sites, the phosphorylated peptide binding the canonical phosphobinding groove of d14‐3‐3ζ. While binding of SP‐(432–439) to d14‐3‐3ζ is similar to other phosphopeptides, the binding of UP‐(333–362) differs from other unphosphorylated peptides (Fig. 9). Up to now, only a few non‐phosphorylated 14‐3‐3 binding partners have been identified and no consensus binding motif could be found (for review, see [64]). The sequence of UP‐(333–362) identified in our study differs from other peptides so far published. Also, the binding site of unphosphorylated MTBD of MAP2c differs from the previously determined sites for the R18 peptide of Raf, the ExoenzymeS or the glycosylated Ser‐O‐GlcNAc peptide [34, 65, 66, 67] (Fig. 9).

**Fig. 11:**
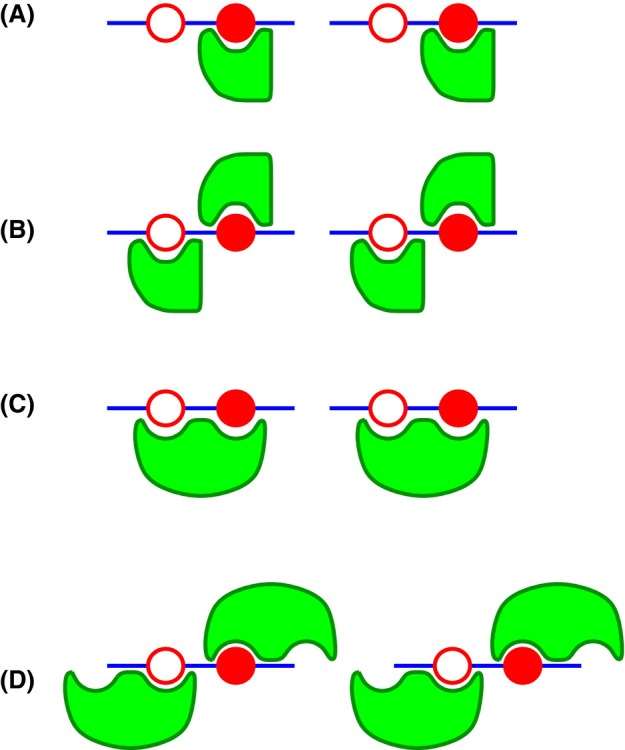
Modes of interaction of a divalent protein ligand with a monomeric and dimeric protein receptor. (A) A monomeric receptor interacting with one ligand site only, resulting in 1 : 1 stoichiometry. (B) A monomeric receptor interacting with each ligand binding site independently, resulting in 1 : 2 stoichiometry. (C) Each molecule of a dimeric receptor interacting with both binding sites of the ligand molecule, resulting in 1 : 2 stoichiometry (per receptor protomer). (D) A dimeric receptor interacting with the ligand binding sites independently, resulting in 1 : 4 stoichiometry (per receptor protomer). The receptor protein is shown in green, and the ligand protein is depicted as a blue bar with the high‐ and low‐affinity binding sites shown as filled and open red circles, respectively.

**Fig. 12:**
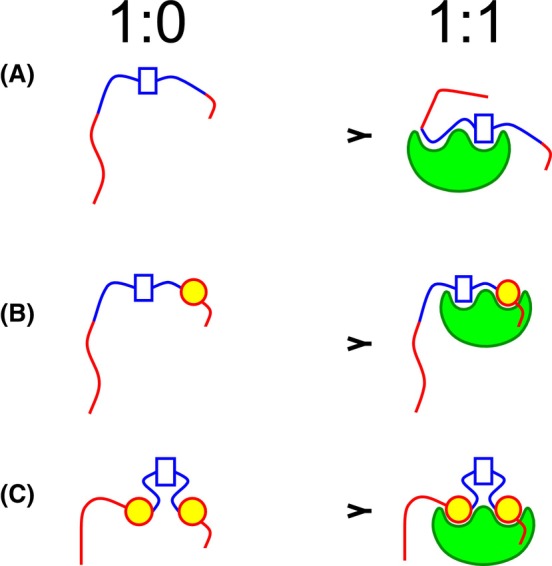
Simplified model of the interaction of MAP2c with dimeric 14‐3‐3ζ (d14‐3‐3ζ). (A) Unphosphorylated MAP2c (UP‐MAP2c), (B) MAP2c selectively phosphorylated by cAMP‐dependent protein kinase (PKA) at Ser435 (SP‐MAP2c), and (C) MAP2c fully phosphorylated by PKA (MP‐MAP2c) with d14‐3‐3ζ. The positively and negatively charged regions of MAP2c are shown in blue and red, respectively, and d14‐3‐3ζ is shown in green. Rectangles represent the unphosphorylated binding site in the microtubule binding domain (MTBD), yellow circles represent phosphorylated binding sites. For the sake of clarity, only one unphosphorylated and two phosphorylated binding sites are depicted for MP‐MAP2c.

The complexity of the interactions of MAP2c with d14‐3‐3ζ and m14‐3‐3ζ is also reflected by the observed apparent stoichiometries. To interpret the obtained results, we first compare different ways in how a multivalent protein ligand can theoretically interact with a monomeric or dimeric protein (called “receptor” for the sake of brevity) presenting one binding site per protomer (Fig. 11). The 1 : 1 stoichiometry (*n* = 1) is observed if only one molecule of a monomeric receptor can interact with the ligand, i.e., if only one binding site of the ligand has a sufficient affinity at the given conditions (Fig. 11A). Higher *n* is obtained if more molecules of the monomeric receptor can interact with the multivalent ligand (shown in Fig. 11B for two binding sites and *n* = 2). The 1 : 2 stoichiometry (*n* = 2) is observed if a dimeric receptor binds two sites of the ligand so that both sites of the receptor are occupied (Fig. 11C). Higher *n* is observed if each molecule of the dimeric receptor preferentially binds the high‐affinity ligand site, and interacts with the low‐affinity site at a high receptor concentration (Fig. 11D). Note that such multivalent binding may result in the formation of larger complexes involving interactions of one dimeric receptor with two ligands, not shown in Fig. 11. Such interactions would result in *n* = 2 at high receptor concentrations. Comparison of the binding modes presented in Fig. 11 with the overall stoichiometries determined experimentally shows that each d14‐3‐3ζ dimer interacts with one UP‐MAP2c or MP‐MAP2c molecule (*n* = 2). A similar avidity effect was observed for doubly phosphorylated fragment from human tyrosine hydroxylase with respect to their singly phosphorylated variants [68]. A higher *n* was observed only for SP‐MAP2c. Its origin may be a presence of a single high‐affinity site in SP‐MAP2c. Interactions of additional weaker binding sites do not influence the overall affinity substantially. In the case of the m14‐3‐3ζ complexes with SP‐MAP2c and MP‐MAP2c, *n* closer to 1 suggests preferred formation of the 1 : 1 complexes (Fig. 11A), with some contribution of the binding modes presented in Fig. 11B and/or Fig. 11C (the latter case involving partial dimerization of m14‐3‐3ζ at high concentrations used in the ITC measurements). Combination of the obtained data allowed us to propose the following model for the MAP2c:d14‐3‐3ζ binding. (a) Several regions of UP‐MAP2c are able to bind d14‐3‐3ζ. The X‐ray structure clarified that MTBD binds to the same pocket as phosphopeptides, but interacts with different d14‐3‐3ζ residues. Different binding regions of MAP2c are sufficiently far in the sequence so that they occupy different pockets of d14‐3‐3ζ simultaneously. (b) In the case of MAP2c phosphorylated on pSer435, the phosphorylated binding site occupies the phosphobinding groove on one monomer, while the MTBD can still interact with the binding site on the other monomer. (c) Upon phosphorylation of more binding sites by PKA, the higher affinity of phosphorylated binding sites results in their binding to the phosphobinding grooves of both monomers, blocking the access of unphosphorylated binding sites to the 14‐3‐3ζ interaction site depicted in Fig. 8E by steric hindrance. The model of the interactions is presented schematically in Fig. 12. However, note that the proposed model represents only a simplified picture of the MAP2c:d14‐3‐3ζ interactions, trying to capture its most significant features.

As discussed above, phosphorylation by PKA does not prevent the d14‐3‐3ζ binding to MTBD but targets d14‐3‐3ζ to other binding sites on MP‐MAP2c. The interactions enhanced by PKA can be further modulated, e.g., by proline‐directed kinases active in the brain. ERK2 phosphorylation has only a minor effect on the interaction of MAP2c with d14‐3‐3ζ. Yet, phosphorylation of residues surrounding pSer435 by ERK2 (pSer422, pSer430, and pSer448) leads to a decrease of affinity in the vicinity of pSer435 to d14‐3‐3ζ and an increase of affinity of the MTBD. A decrease in the affinity for the main phospho‐binding site pSer356 has been observed in hyperphosphorylated Tau, due to the preferential binding to the new binding site created by phosphorylation of pSer352 in hyperphosphorylated Tau [55]. Phosphorylation of MAP2c by ERK2 does not create a new binding site for d14‐3‐3ζ, and the decrease of affinity for pSer435 may be rather explained by electrostatic repulsion between d14‐3‐3ζ and the negatively charged region in CTD created upon ERK2 phosphorylation (Fig. 6). It suggests a possible cross‐talk between the regulation of the MAP2c function by PKA and ERK2. Ser435 seems to be well suited for such fine‐tuning. Ser435 is the first residue phosphorylated by PKA *in vitro* and in the neuroblastoma cell extracts [21]. Also, pSer435 is dephosphorylated slowly in the neuroblastoma cell extract [13]. The biological role of the CTD of MAP2c and of its interactions is poorly understood, but its efficient phosphorylation by PKA suggests an important regulatory function. Ser422 and pSer430, phosphorylated by ERK2 and preceding the major PKA phosphorylation site pSer435, are located in the sequence _417_EIITQSPSRSSVASPRR_433_, homologous with the binding site for cholinergic muscarinic receptors M1 and M3 in Tau [69]. Phosphorylation of serines corresponding to Ser422 or Ser435 decreases microtubule binding [70]. Interestingly, serine corresponding to Ser422 in our sequence is hyperphosphorylated in schizophrenic patients, and knockout 14‐3‐3ζ mice exhibit schizophrenia‐like phenotype [71, 72]. It suggests that the interaction of MAP2c with 14‐3‐3ζ may play a role in schizophrenia. Further biological studies are needed to clarify the role of this sequence in MAP2c. If a therapeutic potential of an intervention of the MAP2c binding to 14‐3‐3ζ is revealed, our structural data may guide the development of small‐molecule inhibitors of the interaction.

We also investigated interactions of MAP2c with the monomeric form of 14‐3‐3ζ. PKA, among other kinases, phosphorylates 14‐3‐3ζ on Ser58 *in vivo*, leading to dissociation of the dimer [73]. In our study, we used the 14‐3‐3ζ L12E/M78K mutant m14‐3‐3ζ present mostly in a monomeric form [41, 42, 74], to mimic the monomerization upon Ser58 phosphorylation. Although the binding groove of m14‐3‐3ζ does not differ from d14‐3‐3ζ, we observed remarkable differences in the interactions with MAP2c, most likely due to the absence of the avidity effect. Interestingly, m14‐3‐3ζ binds only the phosphorylated residues of MAP2c. Monomerization of 14‐3‐3ζ thus leads to the loss of affinity of m14‐3‐3ζ for the MTBD of MAP2c, contrary to pTau [55]. On the other hand, m14‐3‐3ζ interacts with MP‐MAP2c with a similar affinity as d14‐3‐3ζ. This affinity is similar to the affinity of pTau for d14‐3‐3ζ, while affinity of pTau for m14‐3‐3ζ is 10 times lower, in hundreds of μm [55]. The residues involved in the interaction are pSer184, pThr220, pSer435, and the whole C‐terminus of MAP2c. The presence of the single binding groove results in a simple monovalent binding, unless m14‐3‐3ζ dimerization upon MAP2c binding occurs.

The observed differences between MAP2c binding to d14‐3‐3ζ and m14‐3‐3ζ suggest that the formation of the 14‐3‐3ζ dimer stabilizes the binding site of unphosphorylated residues. Binding of the unphosphorylated residues cannot rely on electrostatic interactions of the phosphorylated residues responsible for binding the phosphopeptides. As shown in Fig. 6, the electrostatic potential around the unphosphorylated interaction sites in VCR and especially in MTBD is positive.

In conclusion, we have shown that unphosphorylated MAP2c binds d14‐3‐3ζ via its PRR and MTBD. Phosphorylation of MAP2c by PKA targets d14‐3‐3ζ to phosphorylated residues on MP‐MAP2c, in CTD and PRR, releasing the MTBD and therefore not interfering with microtubules binding, contrary to Tau. The crystal structure of d14‐3‐3ζ with MAP2c peptides shows that phosphorylated and unphosphorylated residues occupy different binding sites on d14‐3‐3ζ. On the other hand, monomerization of 14– 3–3ζ leads to a loss of affinity for unphosphorylated MAP2c. Our study suggests a new mode of interaction of MAP2c with 14‐3‐3ζ, different from its interaction with Tau.

## Materials and methods

### Protein expression and purification

MAP2c fragments consisting of residues 300–399, referred to as UP‐(300–399), and 300–467 were amplified from rat MAP2c in pET3d using the primers 5′‐atcatgccatggctcttcggctcattaaccaacctc, 5′‐attcgcggatcctcagctgtcaatcttcacgttaccacc, and 5′‐attcgcggatccttacaagccctgcttagcgagcg and cloned into pET28a vector using NcoI and BamHI restriction sites. Rat MAP2c wild‐type (UniProt ID A6KFC7), MAP2c‐T220E, and MAP2c‐S435D (residues 1–467) expression and purification were performed as described earlier [1, 13, 21, 75]. The same protocol was used for expression and purification of the MAP2c fragments 300–399 and 300–467. [^15^N]‐MAP2c, [^15^N,^13^C]‐MAP2c, and [^15^N,^13^C]‐UP‐(300–399) were expressed in M9 medium containing 1 g·L^−1^ [^15^N]H_4_Cl and 2 g·L^−1^ [^13^C_6_]glucose (Cambridge Isotope Laboratory, Tewksbury, MA, USA) as the sole sources of nitrogen and carbon, respectively. The dimeric 14‐3‐3ζ (residues 1–245, UniProt Code P63104) contains the additional SVD residues in N‐terminal. Two cysteines Cys25 and Cys189 located on the surface were mutated to alanines to prevent the formation of intermolecular disulfide bridges. The dimeric pseudo wild‐type 14‐3‐3ζ (14‐3‐3ζ C25A/C189A) was expressed and purified as described before [68]. Expression and purification of the monomeric 14‐3‐3ζ‐L12E/M78K/C25A/C189A was described previously [41].

ERK2 and MEKR were purified as described previously [76]. The catalytic domain of PKA cloned in pET15b was expressed in *Escherichia coli* BL21(DE3)RIPL at 30 °C for 4 h. The PKA catalytic subunit was then purified on HisTrap HP column (GE Healthcare, Chicago, IL, USA) in 20 mm Tris‐HCl, 500 mm NaCl, 20 mm imidazole, and eluted with a 20–300 mm imidazole gradient. The eluted fractions were dialyzed in 20 mm Tris‐HCl, pH 8 and further purified on HiTrap Q column (GE Healthcare) in 20 mm Tris, pH 8 and eluted with a step gradient of 0–500 mm NaCl.

Common chemicals were obtained from Carl Roth (Karlsruhe, Germany) and Penta (Praha, Czech Republic).

### Protein phosphorylation

MAP2c and the MAP2c fragment 300–467 were phosphorylated by PKA using 4 μg·mL^−1^ PKA. MP‐MAP2c and SP‐(300–467) samples were prepared by incubating MAP2c and the fragment 300–467 overnight with PKA at 27 °C while MAP2c phosphorylated only on Ser435 (SP‐MAP2c) was prepared by incubating MAP2c with PKA for 1 h at 27 °C, and PKA was deactivated by incubation for 15 min at 75 °C. MAP2c was phosphorylated by ERK2 by mixing MAP2c, ERK2 (60 μg·mL^−1^), and MEKR (20 μg·mL^−1^), and incubated overnight at 27 °C. MAP2c phosphorylated by PKA and ERK2 was first phosphorylated by PKA overnight, the sample was then heated at 75 °C for 15 min to deactivate PKA, and ERK2 and MEKR were then added. All phosphorylation reactions were done in 50 mm MOPS, 100 mm NaCl, 0.5 mm TCEP, 10 mm ATP, 10 mm MgCl_2_,0.1 mm EDTA, at 27 °C for 20 h. Phosphorylated MAP2c was further purified on HiTrap SP column after phosphorylation.

### Isothermal titration calorimetry

For ITC experiments, MAP2c and d14‐3‐3ζ and m14‐3‐3ζ were dialyzed overnight in 50 mm MOPS, 100 mm NaCl, 4 mm β‐mercaptoethanol. Protein concentration was determined by reading optical density at 280 nm. As MAP2c contains only one Trp and five Tyr residues, and impurities may interfere with reading the absorbance at 280 nm, we adjusted the concentration by recording 1D NMR spectra of MAP2c and measuring the area of methyl peaks (1.0–0.0 p.p.m.) and comparing it with the area of methyl peaks of 1 mm ubiquitin (Asla Biotech, Riga, Latvia). The peptides were obtained in powder and diluted in water to 6.5 mg·mL^−1^ SP‐(432–439) and 9.5 mg·mL^−1^ UP‐(333–362), and the concentration was confirmed using 1D NMR as described. Binding of MAP2c to 14‐3‐3 was carried out at 27 °C using Auto PEAQ‐ITC (Malvern Instruments, Malvern, UK). Two microliters of d14‐3‐3ζ or m14‐3‐3ζ were injected with a microsyringe into the 340 μL calorimeter cell containing UP‐MAP2c, SP‐MAP2c, or MP‐MAP2c at 90 μm concentration, 160 μm SP‐(432–439), 150 μm UP‐(333–362), and 220 μm UP‐(300–467) and SP‐(300–467) to achieve a complete binding isotherm. The concentration of d14‐3‐3ζ was 2.9 mm for UP‐MAP2c and MP‐MAP2c and 3 mm for SP‐MAP2c, SP‐(432–439), UP‐(333–362), UP‐(300–467), and SP‐(300–467). The concentration of m14‐3‐3ζ was 1.5 mm in all measurements. The heat of dilution was measured by injecting the d14‐3‐3ζ or m14‐3‐3ζ into the buffer solution. Blank measurement was measured by titrating the buffer into the cell containing the protein of interest. The heat of dilution and the blank measurement were subtracted from the heat of the reaction to obtain the effective heat of binding. Titration curves were fitted using the microcal peaq‐itc software, Malvern Panalytical, Malvern, United Kingdom, assuming one set of sites. In the case of UP‐(333–362) and SP‐(432–439) peptides that bind too weakly to determine the stoichiometry, *n* was set to one during the fitting.

### Nuclear magnetic resonance

Proteins were dialyzed in NMR buffer (50 mm MOPS, 100 mm NaCl, 0.5 mm TCEP) before NMR measurement. HSQC and HNCO spectra were acquired on an 850 and 950 MHz Bruker Avance III spectrometer, Bruker BioSpin GmbH, Rheinstetten, Germany equipped with TCI cryogenic probe heads with *z*‐axis gradients. All experiments were performed at 27 °C with the temperature calibrated according to the chemical shift differences of pure methanol peaks. The ^1^H,^15^N‐HSQC [77, 78] spectra were recorded with spectral widths set to 11 904 Hz in the direct dimension and to 2240 Hz in the indirect dimension, acquiring 2048 and 256 complex points in the direct and the indirect dimensions, respectively. The indirect dimensions in 3D experiments were acquired in a non‐uniformly sampled manner. On‐grid Poisson disk sampling with a Gaussian probability distribution [79] was applied. The 3D HNCO spectra were acquired as in [21]. The HNCO experiment [80] was performed with spectral widths set to 18 939 (aq) × 2000 (^15^N) × 2000 (^13^C′) Hz and maximal evolution times of 120 ms for ^15^N and 80 ms for ^13^C′ indirectly detected dimensions. The overall number of 2048 complex points was acquired in the acquisition dimension and 2000 hypercomplex points were randomly distributed over the indirectly‐detected dimensions. The ^1^H, ^15^N‐HSQC [77, 78] spectra were recorded with spectral widths set to 11 904 Hz in the direct dimension and to 2240 Hz in the indirect dimension. 2048 and 256 complex points were acquired in the direct and the indirect dimensions, respectively.

The uniformly sampled data processing and direct dimension processing of non‐uniformly sampled data were done using nmrpipe software [81]. The Multidimensional Fourier Transform with iterative algorithm for artifact suppression [82] was employed to process indirect dimensions in three‐dimensional experiments. Spectra analysis was done using software sparky 3.115 (T.D. Goddard and D. G. Kneller, University of California, San Francisco, CA, USA).

^31^P NMR spectra were acquired on a 500 MHz spectrometer Bruker Avance NEO equipped with a 5 mm nitrogen‐cooled dual (BB‐1H) cryoprobe (Prodigy) using presaturation. The α‐ATP signal at δ = −10.03 p.p.m. was used as chemical shift reference [83].

### Identification of binding sites in MAP2c

The interactions of [^15^N,^13^C]‐UP‐MAP2c, [^15^N,^13^C]‐SP‐MAP2c, [^15^N,^13^C]‐MP‐MAP2c, ERK2‐phosphorylated [^15^N,^13^C]‐MAP2c, [^15^N,^13^C]‐MAP2c phosphorylated by PKA and ERK2, and [^15^N,^13^C]‐UP‐(300–399) were monitored in 3D HNCO spectra. All proteins were dialyzed in NMR buffer. Titration of MP‐MAP2c with 14‐3‐3ζ was done by mixing 100 μm [^13^C,^15^N]‐MP‐MAP2c with 12.5, 25, 50, 100 and 200 μm d14‐3‐3ζ or m14‐3‐3ζ, respectively. To identify interacting sites in other MAP2c samples, 100 μm [^15^N,^13^C]‐MAP2c samples were mixed with 200 μm d14‐3‐3ζ or m14‐3‐3ζ. HNCO spectra of UP‐MAP2c were also acquired at 100 μm d14‐3‐3ζ and m14‐3‐3ζ concentrations. The interacting residues in MTBD were monitored by mixing 350 μm [^15^N,^13^C]‐UP‐(300–399) with 350 μm d14‐3‐3ζ.

Titration of ^31^P peaks with d14‐3‐3ζ and m14‐3‐3ζ were monitored by mixing 60 μm SP‐MAP2c or MP‐MAP2c with 12.5, 25, 50, 100, 200, and 300 μm d14‐3‐3ζ or 12.5, 25, 50, 100, 200 μm m14‐3‐3ζ, respectively. The peak areas were fitted using Eqn (1), where *x* is the frequency, *f* (*x*) is the signal intensity, *p* is the maximum peak height, and *m* is the position (chemical shift) of the peak maximum.

(1) $$f\left(x\right)=p\cdot e^{\left(-{\left(x-m\right)}^{2}/\left(2\cdot s^{2}\right)\right)}$$

### Native PAGE

For native PAGE, 100 μm d14‐3‐3ζ or m14‐3‐3ζ were mixed with 0, 50, 100, 200, and 400 μm of MP‐MAP2c and incubated 15 min at room temperature. Samples were run on 10% polyacrylamide gel for 3 h at 100 V at 4 °C in native running buffer (25 mm tris, 192 mm glycine). The gel was quantified using gelanalyser 19.1.1 software (available at www.gelanalyzer.com by Istvan Lazar Jr., PhD and Istvan Lazar Sr., PhD, CSc).

### Chemical crosslinking

The crosslinking reaction mixtures were prepared by mixing 20 μm UP‐MAP2c or MP‐MAP2c with 80 μm d14‐3‐3ζ or m14‐3‐3ζ, and PBS buffer with 1 mm TCEP. After vortexing, the samples were incubated at 37 °C for 30 min. The crosslinking reactions were initiated by adding 200 μm disuccinimidyl dibutyric urea cross‐linker (DSBU; Thermofisher Scientific, Waltham, MA, USA), and quenched by adding 100 mm Tris after 30 min. After 10 min, 4× loading buffer was added, and samples were placed at 95 °C for 10–15 min. The crosslinking reaction products were analyzed by SDS/PAGE. The size of the bands of crosslinking reactions was determined by linear fit, using the PageRuler Prestained Protein Ladder (Thermofisher Scientific) as standard. For control, 80 μm d14‐3‐3ζ or 20 μm MP‐MAP2c were incubated with DBSU for 15 min. The same conditions were used for crosslinking of d14‐3‐3ζ with UP‐(333–362), using 80 μm d14‐3‐3ζ and 100 or 400 μm UP‐(333–362).

Selected bands were excised from the gels and trypsin‐digested proteins were analyzed on LC‐MS/MS system (RSLCnano connected to Orbitrap Fusion Lumos Tribid; Thermo Fisher Scientific). Raw MS spectra were converted to Mascot generic files (mgf) using ProteoWizard and subsequently analyzed in MeroX for putative cross‐links [55].

### X‐ray crystallography of d14‐3‐3ζ complexes with peptides derived from MAP2c

Purified d14‐3‐3ζ was dialyzed in buffer containing 20 mm Tris‐HCl pH (8.0), 75 mm NaCl, 2 mm NaN_3_. Peptides for X‐ray crystallography were designed based on the sequence of *Rattus norvegicus* MAP2c and ordered from Clonestar, Brno, Czech Republic. The peptide sequences are as follows: SP‐(432–439) [_432_RRL(pS)NVSS_439_] and UP‐(333–362) [_333_QIVTKKIDLSHVTSKCGSLKNIRHRPGGGR_362_]. Initial crystals were grown at 20 °C in hanging drops by vapor diffusion using molar excess of peptides to protein (protein : peptide ratio 1 : 3) at 10 mg·mL^−1^ in 20 mm Tris–HCl (pH 8.0), 50 mm NaCl, 2 mm NaN_3_ with reservoir solution containing 100 mm HEPES (pH 7.5), 400 mm sodium acetate, 10 mm cadmium sulfate. These crystals were substantially improved by microseeding into a pre‐equilibrated solution (protein : peptide solution at 1 : 3 ratio) for 2–3 days made up with a reservoir solution containing 100 mm HEPES (pH 7.5), 400 mm sodium Acetate, 8 mm cadmium sulfate in the case of SP‐(432–439) and 100 mm HEPES (pH 7.5), 600 mm sodium acetate, 6 mm cadmium sulfate in the case of UP‐(333–362). The crystals grew up to typical sizes of 40 × 60 × 60 μm^3^ within 4–6 days. Crystals were prepared for flash cooling by soaking in a cryoprotectant solution made up of 50% glycerol.

### Data collection and structure determination

Diffraction data were collected at the DESY beamline P14 (Hamburg, Germany) at a wavelength of 0.9762 Å with a Dectris EIGER2 CdTe 16M detector. Integrated and scaled data sets from multiple crystals were merged to improve data completeness and redundancy using xscale program within the xds suite [84], the merged data were further processed using aimless [85] to refine scaling parameters and improve data quality. The parameters for merging and scaling were optimized to achieve the best possible merging statistics. Data collection statistics are summarized in Table 3. The data were further processed on ccp4i suite [86]. The data were phased with molrep [87], using PDB ID: 5NAS as the search model. Structure refinement was performed with refmac5 [88] with 5% randomly chosen reflections that were set aside to calculate Rfree. Presence of the ligands was verified by visual inspection of the *F*_o_ − *F*_c_ and 2*F*_o_ − *F*_c_ electron density maps in coot (version 0.9.6) [89]. The model rebuilding and refinement were performed through manual inspection in coot. The images were created using the pymol Molecular Graphic System (Schrödinger LLC, New York, NY, USA version 2.2.3). The hydrogen atoms were added to the peptide structures using the H Add algorithm in pymol (Schrödinger LLC, version 2.2.3). The structures were deposited in the protein data bank (PDB) with ID 9FUM for d14‐3‐3ζ with SP‐(432–439) and 9FVL for d14‐3‐3ζ with UP‐(333–362). Refinement statistics are shown in Table 3.

## Conflict of interest

The authors declare no conflict of interest.

## Author contributions

SJ, SN, JH, and LZ conceived and designed the research; SJ, SN, PCF, AK, and KK prepared the protein samples; LZ, SJ, and PCF performed the NMR experiments; SN determined the crystal structure; LI performed the crosslinking studies; SJ, SN, JH, and LZ wrote the paper. All authors reviewed the results and approved the final version of the manuscript.

### Peer review

The peer review history for this article is available at https://www.webofscience.com/api/gateway/wos/peer‐review/10.1111/febs.17405.

## Supporting information


**Fig. S1.** Results of isothermal titration calorimetry analysis of unphosphorylated MAP2c, MAP2c selectively phosphorylated by cAMP‐dependent protein kinase (PKA) at Ser435, and MAP2c fully phosphorylated by PKA with dimeric 14‐3‐3ζ and monomeric 14‐3‐3ζ.
**Fig. S2.** Dissociation of monomeric 14‐3‐3ζ dimers into monomers measured by isothermal titration calorimetry.
**Fig. S3.** Results of isothermal titration calorimetry analysis of unphosphorylated MAP2c fragment 300–467 and MAP2c fragment 300–467 phosphorylated by cAMP‐dependent protein kinase titrated with dimeric 14‐3‐3ζ.
**Fig. S4.** ^1^H,^15^N‐HSQC spectra of [^1^H,^15^N]‐unphosphorylated MAP2c bound to dimeric 14‐3‐3ζ and monomeric 14‐3‐3ζ.
**Fig. S5.** ^1^H,^15^N‐HSQC spectra of [^1^H,^15^N]‐MAP2c selectively phosphorylated by cAMP‐dependent protein kinase at Ser435 bound to dimeric 14‐3‐3ζ and monomeric 14‐3‐3ζ.
**Fig. S6.** ^1^H,^15^N‐HSQC spectra of [^1^H,^15^N]‐MAP2c fully phosphorylated by cAMP‐dependent protein kinase bound to dimeric 14‐3‐3ζ and monomeric 14‐3‐3ζ.
**Fig. S7.** ^1^H,^15^N‐HSQC spectra of [^1^H,^15^N]‐MAP2c phosphorylated by extracellular signal‐regulated kinase 2 bound to dimeric 14‐3‐3ζ and monomeric 14‐3‐3ζ.
**Fig. S8.** ^1^H,^15^N‐HSQC spectra of [^1^H,^15^N]‐MAP2c phosphorylated by cAMP‐dependent protein kinase, and extracellular signal‐regulated kinase 2 bound to dimeric 14‐3‐3ζ and monomeric 14‐3‐3ζ.
**Fig. S9.** Results of isothermal titration calorimetry analysis of the peptide corresponding to residues 333–362 of MAP2c (UP‐(333–362)) and of the phosphopeptide corresponding to residues 432–439 (SP‐(432–439)) titrated with dimeric 14‐3‐3ζ.

## Data Availability

The structural data that support these findings are openly available in the wwPDB at https://doi.org/10.2210/pdb9FUM/pdb (complex with SP‐(432–439)) and https://doi.org/10.2210/pdb9FVL/pdb (complex with UP‐(333–362)). All other data that support the findings of this study are available in the figures and tables in the manuscript and in the Supporting Information of this article.
