# Supplementary material for: Characterization of multiple binding sites on microtubule associated protein 2c recognized by dimeric and monomeric 14‐3‐3ζ
Source: FEBS J. 2025 Jan 29;292(8):1991–2016. doi: 10.1111/febs.17405 (PMC12001206; doi:10.1111/febs.17405)
Supplement: Supplementary file 1 — Fig. S1. Results of isothermal titration calorimetry analysis of unphosphorylated MAP2c, MAP2c selectively phosphorylated by cAMP‐dependent protein kinase (PKA) at Ser435, and MAP2c fully phosphorylated by PKA with dimeric 14‐3‐3ζ and monomeric 14‐3‐3ζ. Fig. S2. Dissociation of monomeric 14‐3‐3ζ dimers into monomers measured by isothermal titration calorimetry. Fig. S3. Results of isothermal titration calorimetry analysis of unphosphorylated MAP2c fragment 300–467 and MAP2c fragment 300–467 phosphorylated by cAMP‐dependent protein kinase titrated with dimeric 14‐3‐3ζ. Fig. S4. 1H,15N‐HSQC spectra of [1H,15N]‐unphosphorylated MAP2c bound to dimeric 14‐3‐3ζ and monomeric 14‐3‐3ζ. Fig. S5. 1H,15N‐HSQC spectra of [1H,15N]‐MAP2c selectively phosphorylated by cAMP‐dependent protein kinase at Ser435 bound to dimeric 14‐3‐3ζ and monomeric 14‐3‐3ζ. Fig. S6. 1H,15N‐HSQC spectra of [1H,15N]‐MAP2c fully phosphorylated by cAMP‐dependent protein kinase bound to dimeric 14‐3‐3ζ and monomeric 14‐3‐3ζ. Fig. S7. 1H,15N‐HSQC spectra of [1H,15N]‐MAP2c phosphorylated by extracellular signal‐regulated kinase 2 bound to dimeric 14‐3‐3ζ and monomeric 14‐3‐3ζ. Fig. S8. 1H,15N‐HSQC spectra of [1H,15N]‐MAP2c phosphorylated by cAMP‐dependent protein kinase, and extracellular signal‐regulated kinase 2 bound to dimeric 14‐3‐3ζ and monomeric 14‐3‐3ζ. Fig. S9. Results of isothermal titration calorimetry analysis of the peptide corresponding to residues 333–362 of MAP2c (UP‐(333–362)) and of the phosphopeptide corresponding to residues 432–439 (SP‐(432–439)) titrated with dimeric 14‐3‐3ζ. [file FEBS-292-1991-s001.pdf]

Supporting Information

Title: Characterization of multiple binding sites on microtubule associated protein 2c recognized by dimeric and monomeric 14–3–3ζ

Authors: Séverine Jansen[1,*], Subhash Narasimhan[1,2,*], Paula Cabre Fernandez[2,3], Lucia Il'kovičová[1,2], Kateřina Bendová[1,2], Aneta Kozeleková[1,2], Kateřina Královà[1], Jozef Hritz[1,4], and Lukáš Žídek[1,2]

Affiliations: [1]Central European Institute of Technology, Masaryk University, Kamenice 5, CZ-625 00, Brno, Czech Republic, [2]National Centre for Biomolecular Research, Faculty of Science, Masaryk University, Kamenice 5, CZ-625 00, Brno, Czech Republic, [3] Research Institute Sant Pau, Sant Quinti 77-79, 08041 Barcelona, Spain, [4] Department of Chemistry, Faculty of Science, Masaryk University, Kamenice 5, CZ-625 00, Brno, Czech Republic

[*] Séverine Jansen and Subhash Narasimhan contributed equally to the work.
To whom correspondence should be addressed: Lukáš Žídek, phone: +420 54949 8393, fax: +420 54949 2556, lzidek@chemi.muni.cz

Running title : Interaction of phosphorylated MAP2c with 14-3-3ζ

Contents

Figure S1	Results of Isothermal Titration Calorimetry analysis of unphosphorylated MAP2c, MAP2c selectively phosphorylated by cAMP-dependent Protein Kinase (PKA) at Ser435 and MAP2c fully phosphorylated by PKA titrated with dimeric 14–3–3ζ and monomeric 14-3-3ζ.
Figure S2	Dissociation of monomeric 14-3-3ζ dimers into monomers.
Figure S3	Results of Isothermal Titration Calorimetry analysis of unphosphorylated MAP2c fragment 300–467 and MAP2c fragment 300–467 phosphorylated by cAMP-dependent Protein Kinase titrated with dimeric 14–3–3ζ.
Figure S4	^{1}H,^{15}N-HSQC spectra of [^{1}H,^{15}N]-MAP2c unphosphorylated bound to dimeric 14–3–3ζ and monomeric 14–3–3ζ.
Figure S5	^{1}H,^{15}N-HSQC spectra of [^{1}H,^{15}N]-MAP2c selectively phosphorylated by cAMP-dependent Protein Kinase at Ser435 bound to dimeric 14–3–3ζ and moneric 14–3–3ζ.
Figure S6	^{1}H,^{15}N-HSQC spectra of [^{1}H,^{15}N]-MAP2c fully phosphorylated by cAMP-dependent Protein Kinase bound to dimeric 14–3–3ζ and monomeric 14–3–3ζ.
Figure S7	^{1}H,^{15}N-HSQC spectra of [^{1}H,^{15}N]-MAP2c phosphorylated by Extracellular Signal-Regulated Kinase 2 bound to dimeric 14-3-3ζ and monomeric 14–3–3ζ.
Figure S8	^{1}H,^{15}N-HSQC spectra of [^{1}H,^{15}N]-MAP2c phosphorylated by cAMP-dependent Protein Kinase and Extracellular Signal-Regulated Kinase 2 bound to dimeric 14–3–3ζ and monomeric 14–3–3ζ.
Figure S9	Results of Isothermal Titration Calorimetry analysis of the peptide corresponding to residues 333-362 of MAP2c (UP-(333–362)) and of the phosphopeptide corresponding to residues 432–439 (SP-(432–439)) titrated with dimeric 14–3–3ζ.

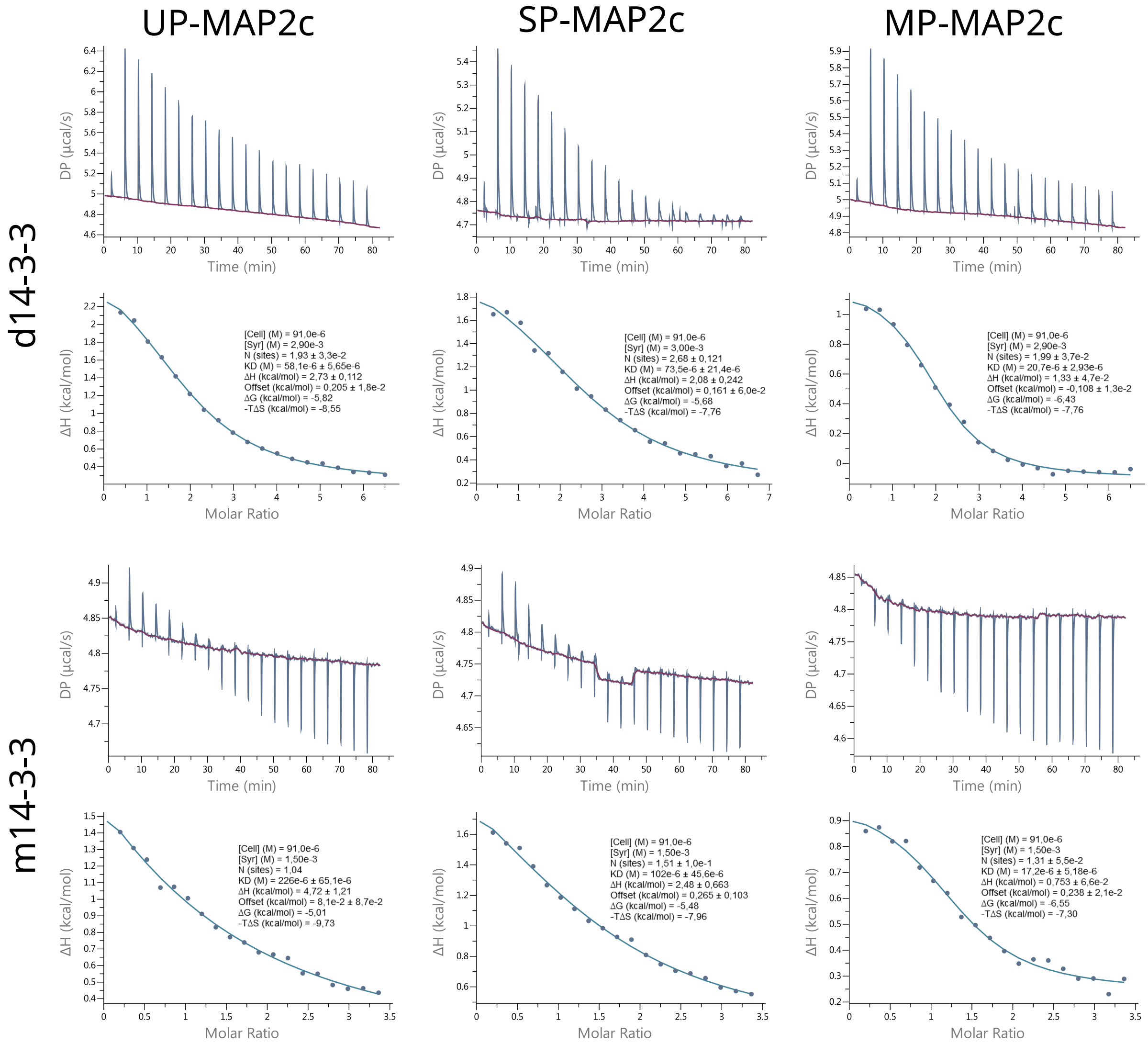

Figure S1: **Results of Isothermal Titration Calorimetry (ITC) analysis of unphosphorylated MAP2c (UP-MAP2c), MAP2c selectively phosphorylated by cAMP-dependent Protein Kinase (PKA) at Ser435 (SP-MAP2c) and MAP2c fully phosphorylated by PKA (MP-MAP2c) titrated with dimeric 14-3-3ζ (d14–3–3ζ) and monomeric 14-3-3ζ (m14–3–3ζ.)** The concentration in the cell was 90 μM and the concentration in the syringe was 2.9 mM and 1.5 mM for d14–3–3ζ and m14–3–3ζ, respectively. ITC was obtained as triplicates for UP-MAP2c and MP-MAP2c with d14-3-3ζ and m14-3-3ζ, only one set of data is shown. For SP-MAP2c, the results are in agreement with NMR data and were not duplicated.

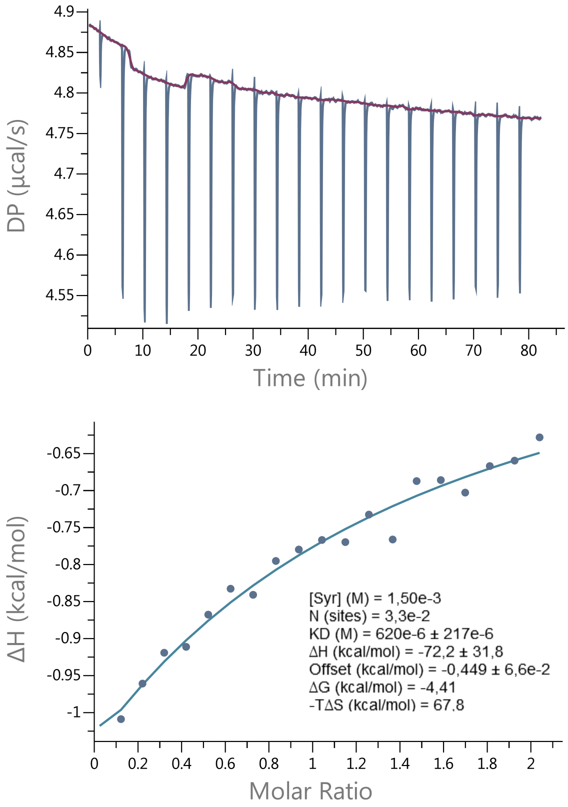

Figure S2: **Dissociation of monomeric 14-3-3ζ (m14-3-3ζ) dimers into monomers**. Isothermal Titration Calorimetry (ITC) titration of m14-3-3ζ at 1.5 mM into ITC buffer, showing the dissociation of m14–3–3ζ at high concentration into monomers. The estimated K_D is (620 ± 217) μM. This auxiliary experiment was performed once.

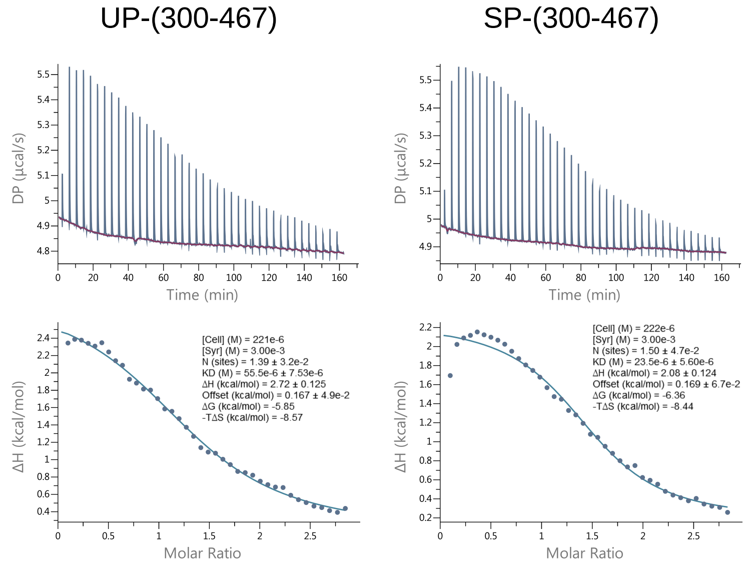

Figure S3: **Results of Isothermal Titration Calorimetry (ITC) analysis of unphosphorylated MAP2c fragment 300–467 (UP-(300–467)) and MAP2c fragment 300–467 phosphorylated by cAMP-dependent Protein Kinase (SP-(300–467)) titrated with dimeric 14–3–3ζ (d14–3–3ζ).** The concentration in the cell was 220 μM for UP-(300–467) and SP-(300–467), and the concentration in the syringe was 3 mM for d14–3–3ζ. ITC results were in agreement with the NMR data, showing similar results.

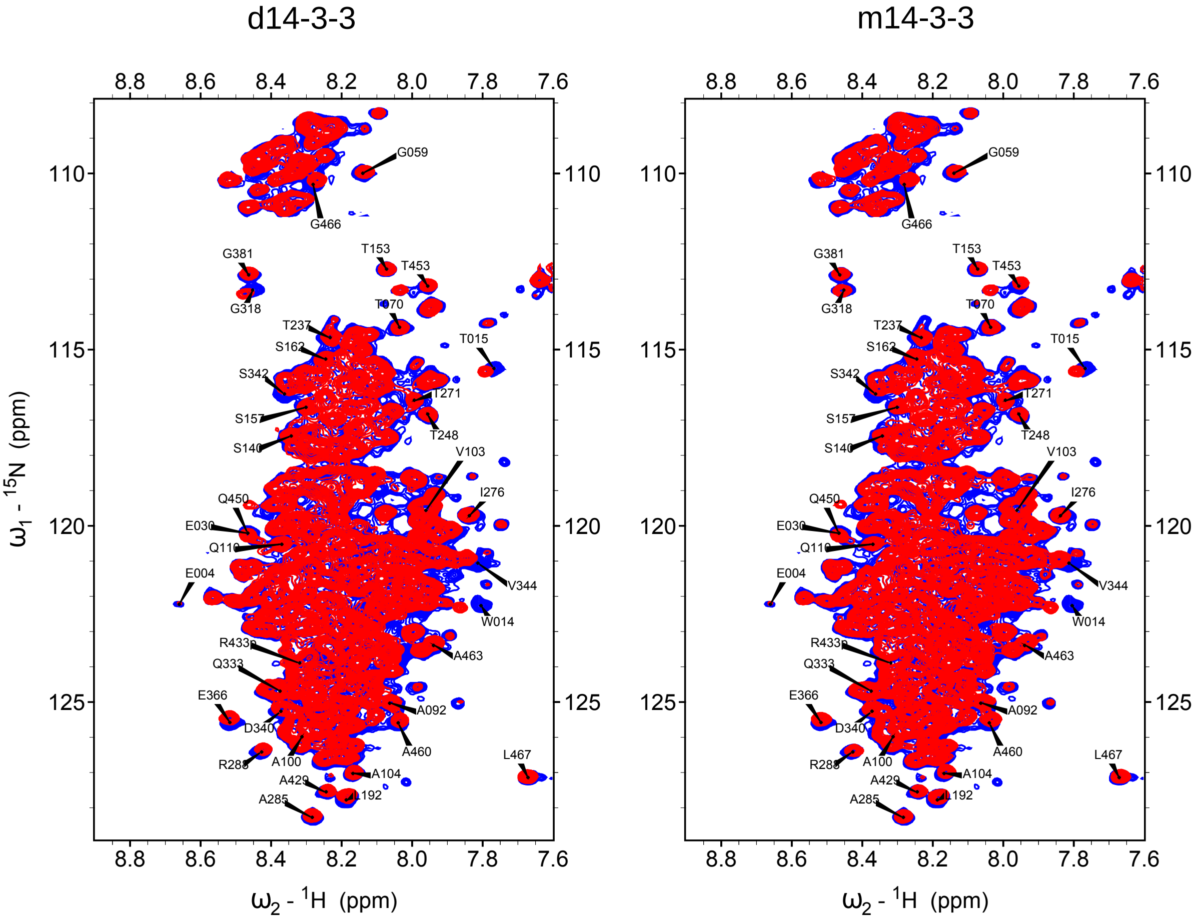

Figure S4: **^{1}H,^{15}N-HSQC spectra of [^{1}H,^{15}N]-MAP2c unphosphorylated (UP-MAP2c) bound to dimeric 14–3–3ζ (d14–3–3ζ) and monomeric 14–3–3ζ (m14–3–3ζ).** Blue, free UP-MAP2c; red, UP-MAP2c bound to d14–3–3ζ (left) or m14–3–3ζ (right). Reproducibility of the spectra was confirmed by comparison with independent HNCO experiments.

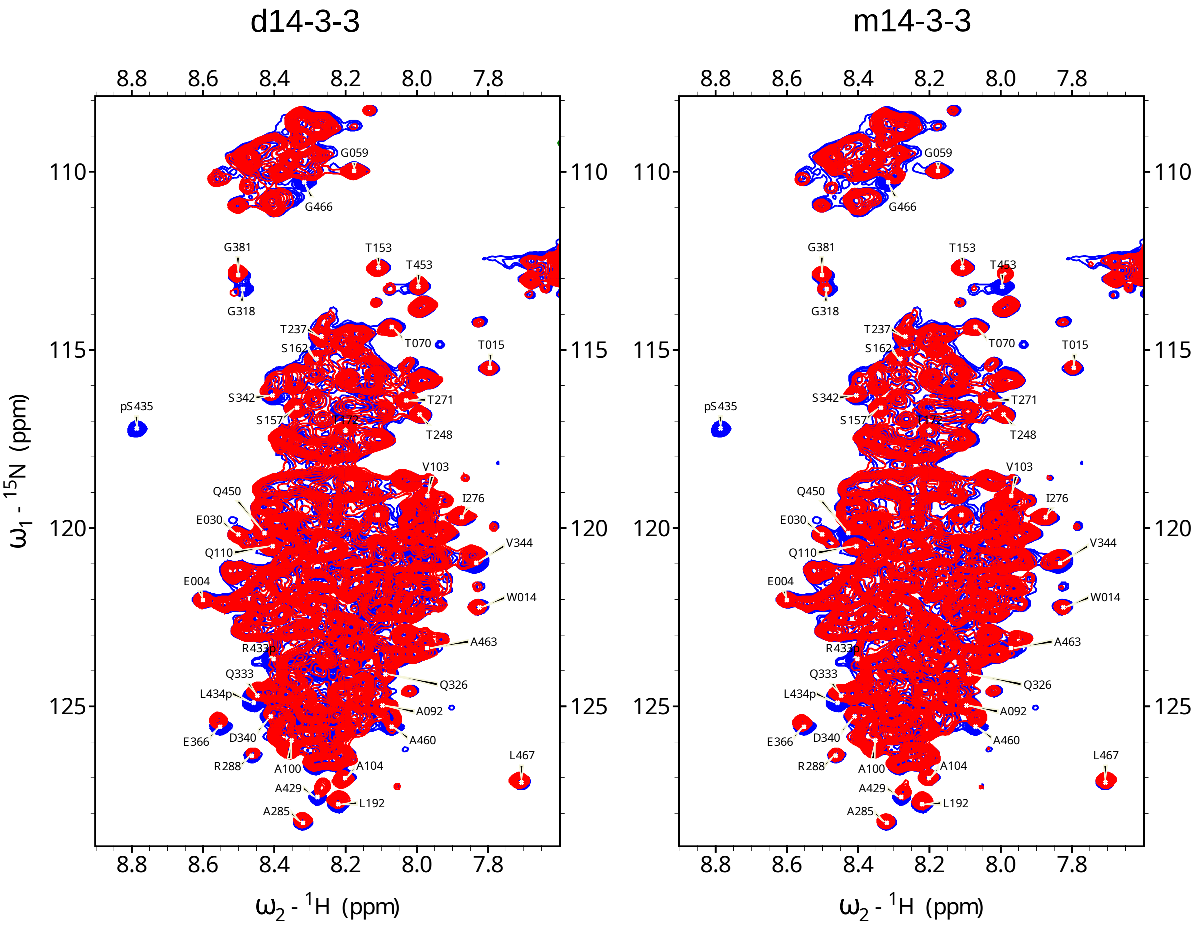

Figure S5: **^{1}H,^{15}N-HSQC spectra of [^{1}H,^{15}N]-MAP2c selectively phosphorylated by cAMP-dependent Protein Kinase (PKA) at Ser435(SP-MAP2c) bound to dimeric 14–3–3ζ (d14–3–3ζ) and monomeric 14–3–3ζ (m14–3–3ζ).** Blue, free SP-MAP2c; red, SP-MAP2c bound to d14–3–3ζ (left) or m14–3–3ζ (right). Reproducibility of the spectra was confirmed by comparison with independent HNCO experiments.

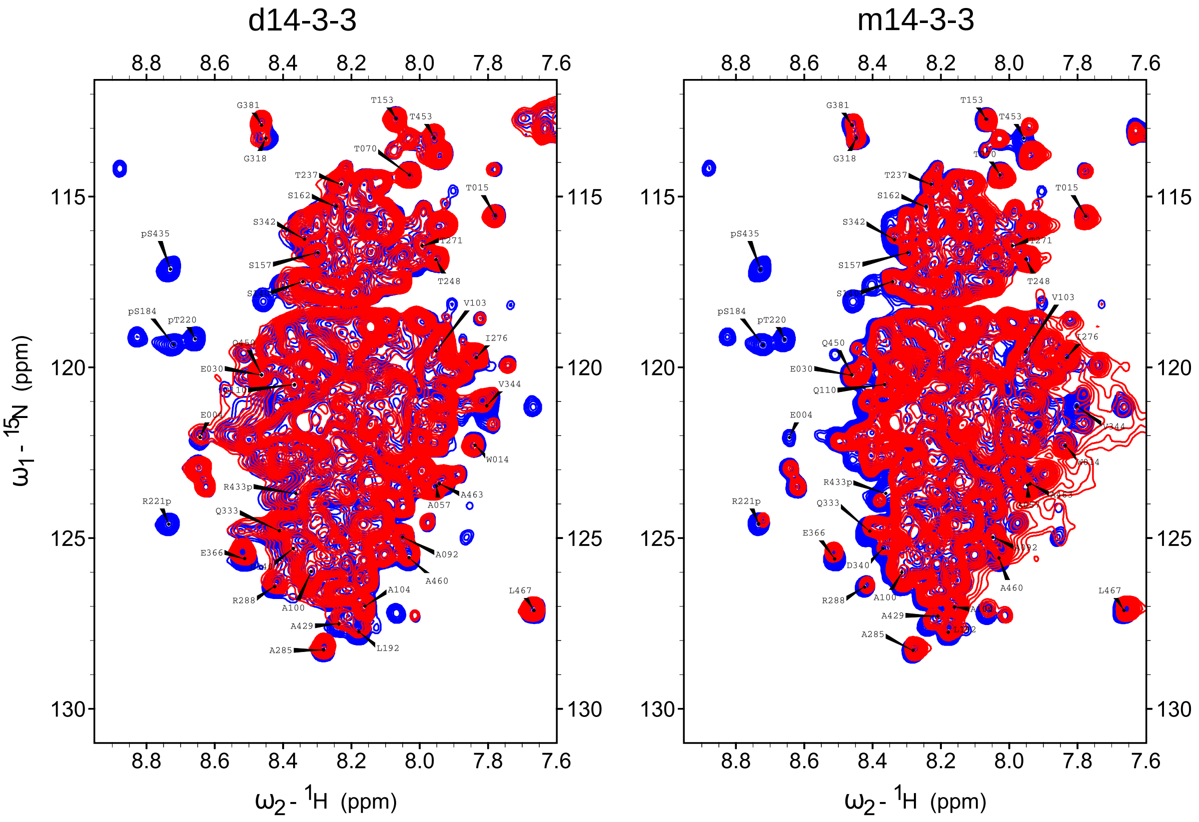

Figure S6: **^{1}H,^{15}N-HSQC spectra of [^{1}H,^{15}N]-MAP2c fully phosphorylated by cAMP-dependent Protein Kinase (MP-MAP2c) bound to dimeric 14–3–3ζ (d14–3–3ζ) and monomeric 14–3–3ζ (m14–3–3ζ).** Blue, free MP-MAP2c; red, MP-MAP2c bound to d14–3–3ζ (left) or m14–3–3ζ (right). Reproducibility of the spectra was confirmed by comparison with independent HNCO experiments.

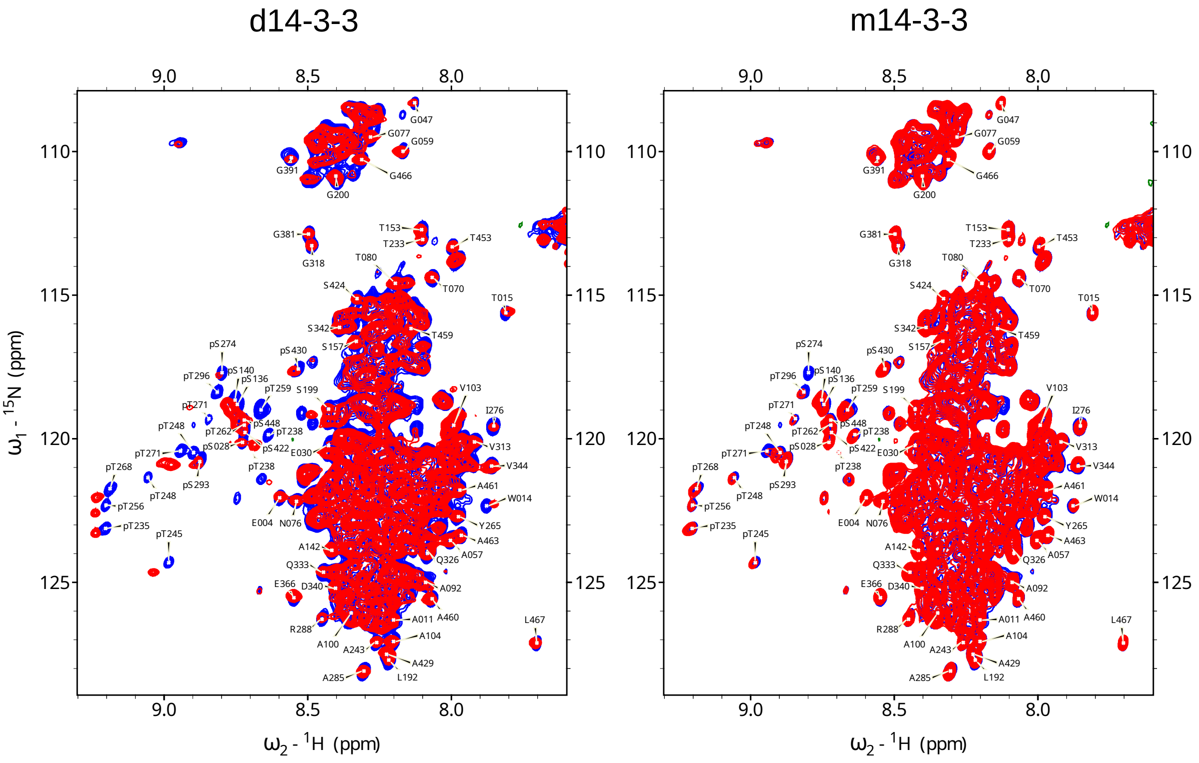

Figure S7: ^{1}H,^{15}N-HSQC spectra of [^{1}H,^{15}N]-MAP2c phosphorylated by Extracellular Signal-Regulated Kinase 2 (ERK2) bound to dimeric 14-3-3ζ (d14–3–3ζ) and monomeric 14–3–3ζ (m14–3–3ζ). Free MAP2c phosphorylated by ERK2 (blue) and bound to d14–3–3ζ (red, left) or m14–3–3ζ (red, right). Reproducibility of the spectra was confirmed by comparison with independant HNCO experiments.

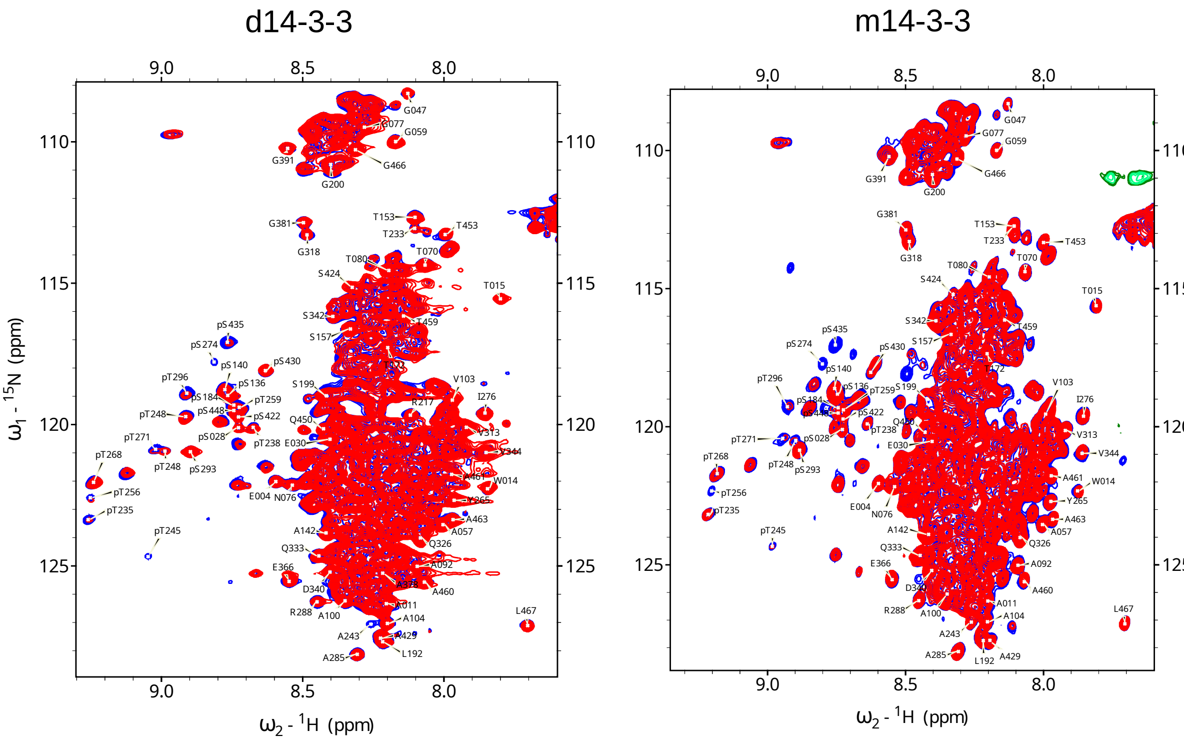

Figure S8: **^{1}H,^{15}N-HSQC spectra of [^{1}H,^{15}N]-MAP2c phosphorylated by cAMP-dependent Protein Kinase (PKA) and Extracellular Signal-Regulated Kinase 2 (ERK2) bound to dimeric 14–3–3ζ (d14–3–3ζ) and monomeric 14–3–3ζ (m14–3–3ζ).** Free MAP2c phosphorylated by PKA and ERK2 phosphorylated (blue) and bound to d14–3–3ζ (red, left) or m14–3–3ζ (red, right). Reproducibility of the spectra was confirmed by comparison with independant HNCO experiments.

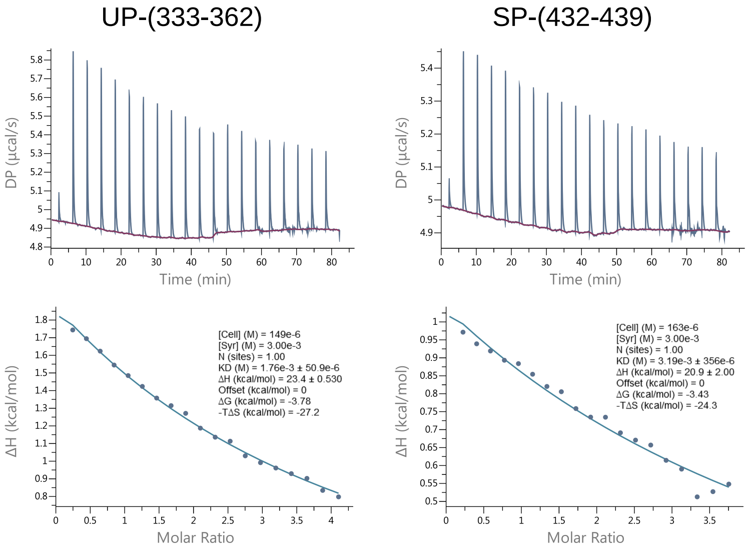

Figure S9: **Results of Isothermal Titration Calorimetry (ITC) analysis of the peptide corresponding to residues 333-362 of MAP2c (UP-(333–362)) and of the phosphopeptide corresponding to residues 432-439 (SP-(432–439)) titrated with dimeric 14–3–3ζ (d14–3–3ζ).** The concentration in the cell was 150 μM and 160μM for UP-(333–362) and SP-(432–439), respectively, and the concentration in the syringe was 3 mM for d14–3–3ζ. As the affinity of the peptides for d14–3–3ζ is too low to determine the number of sites, n was fixed to 1 to calculate the K_{D}. The ITC data were obtained as duplicates, showing similar results. Only one set of data is shown.
